# Rna M^6^a Methylation Regulates Glycolysis of Beige Fat and Contributes to Systemic Metabolic Homeostasis

**DOI:** 10.1002/advs.202300436

**Published:** 2023-07-05

**Authors:** Yu Li, Yankang Zhang, Ting Zhang, Xiaodan Ping, Dongmei Wang, Yanru Chen, Jian Yu, Caizhi Liu, Ziqi Liu, Yuhan Zheng, Yongfeng Yang, Chengchao Ruan, Dali Li, Zhenyu Du, Jiqiu Wang, Lingyan Xu, Xinran Ma

**Affiliations:** ^1^ Shanghai Key Laboratory of Regulatory Biology Institute of Biomedical Sciences and School of Life Sciences East China Normal University Shanghai 200241 China; ^2^ Chongqing Key Laboratory of Precision Optics Chongqing Institute of East China Normal University Chongqing 401120 China; ^3^ Department of Endocrinology and Metabolism Ruijin Hospital Shanghai Jiao Tong University School of Medicine Shanghai 200025 China; ^4^ Department of Endocrinology and Metabolism Fengxian Central Hospital Affiliated to Southern Medical University Shanghai 201499 China; ^5^ Department of Physiology and Pathophysiology School of Basic Medical Sciences Fudan University Shanghai 200032 China; ^6^ Shanghai Frontiers Science Center of Genome Editing and Cell Therapy Shanghai Key Laboratory of Regulatory Biology and School of Life Sciences East China Normal University Shanghai 200241 China

**Keywords:** beige fat, energy homeostasis, glycolysis, N6‐methyladenosine, Mettl3, preadipocytes proliferation

## Abstract

N6‐methyladenosine (m^6^A) modification has been implicated in the progression of obesity and metabolic diseases. However, its impact on beige fat biology is not well understood. Here, via m^6^A‐sequencing and RNA‐sequencing, this work reports that upon beige adipocytes activation, glycolytic genes undergo major events of m^6^A modification and transcriptional activation. Genetic ablation of m^6^A writer Mettl3 in fat tissues reveals that Mettl3 deficiency in mature beige adipocytes leads to suppressed glycolytic capability and thermogenesis, as well as reduced preadipocytes proliferation via glycolytic product lactate. In addition, specific modulation of Mettl3 in beige fat via AAV delivery demonstrates consistently Mettl3's role in glucose metabolism, thermogenesis, and beige fat hyperplasia. Mechanistically, Mettl3 and m^6^A reader Igf2bp2 control mRNA stability of key glycolytic genes in beige adipocytes. Overall, these findings highlight the significance of m^6^A on fat biology and systemic energy homeostasis.

## Graphical abstract

N6‐methyladenosine (m^6^A)‐Seq and RNA‐Seq reveal the role of RNA m^6^A modification in beige fat glycolysis upon activation. Ablation of m^6^A writer Mettl3 in beige fat suppresses glycolytic capability, thermogenesis, and hyperplasia. Mechanistically, Mettl3 and m^6^A reader Igf2bp2 control mRNA stability of key glycolytic genes in beige adipocytes, suggesting the significance of m^6^A on fat biology and systemic energy homeostasis.

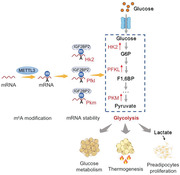

## 1 Introduction

Obesity manifests as an excess accumulation of body fat and is a major risk factor for metabolic syndromes, including type 2 diabetes, hepatic steatosis, and cardiovascular diseases.^[^ ^1^ ^]^ Adipose tissues, including white, brown, and beige fat, play critical roles in maintenance of metabolic homeostasis with their potential of expanding and remodeling upon a wide range of metabolic challenges to increase the adipocyte size, number, and activity, thus regulates energy storage or utilization.^[^ ^2^, ^3^ ^]^ Beige fat, for example, is a highly flexible adipose tissue that characterizes significant induction and activation upon temperature changes,^[^ ^4^, ^5^ ^]^ a process referred to as “browning of white fat,” during which they experience marked morphological and functional transitions including multilocular lipid droplets, increased mitochondria contents and enhanced thermogenesis, therefore contributes greatly toward energy dissipation.

Of note, upon activation, beige adipocytes take up considerable amounts of glucose and lipid as energy substrates, thus functions as a metabolic sink to maintain systemic glucose and lipid homeostasis aside from their body weight‐reducing effects. PET‐Scan analysis showed that functional beige adipocytes are present in adult human that actively take up ^18^F‐2‐deoxy‐d‐glucose (FDG) upon activation,^[^ ^6^, ^7^ ^]^ suggesting glucose metabolism is vital for beige fat functionality. Indeed, it has been established that beige adipocytes possess marked glycolytic capability independent of *β*3‐adrenergic signaling, and that restriction of glycolysis significantly impaired energy metabolism, glucose utilization, and oxygen consumption in beige adipocytes.^[^ ^8^, ^9^ ^]^ Furthermore, beige adipocytes have been shown to maintain glucose metabolism and energy expenditure in interscapular BAT‐removal mice, indicating a critical role of beige fat for glucose homeostasis.^[^ ^10^ ^]^

Extensive studies have given us a glimpse of the complexed regulatory network, that is, transcription factors such as Prdm16 and Pgc1*α*, accompanied with epigenetic regulators like Ehmt and Lsd1, which together regulating beige identity and functionality.^[^ ^11^, ^12^, ^13^, ^14^ ^]^ Beyond transcriptional regulation, post‐transcriptional regulation has major impacts on signaling cascade, yet how it influences fuel usage and metabolic homeostasis of beige adipocyte remained largely unknown. N6‐methyladenosine (m^6^A) modification of mRNA represents a main aspect of post‐transcriptional regulation, which is a finely‐tuned and dynamic process that rely on specific classes of regulatory factors, including m^6^A writers (Mettl3, Mettl14, Wtap, etc.), erasers (Alkbh5, Fto etc.), and readers (Ythdc, Ythdf, Igf2bp2, etc.).^[^ ^15^, ^16^, ^17^ ^]^ m^6^A modifications are indispensable for numerous basic biological processes such as cell fate commitment, organ development, cell proliferation and survival, as well as implicated in neurodegenerative diseases, autoimmunity diseases, and many types of cancers.^[^ ^18^, ^19^ ^]^ Intriguingly, m^6^A modification has also been implicated in the progression of metabolic diseases, including obesity, fatty liver, and type 2 diabetes, predominantly through its impacts on fat metabolism or cell proliferation.^[^ ^20^, ^21^, ^22^ ^]^ For example, m^6^A eraser Fto has been shown as one of the leading risk factors for obesity in human and rodents.^[^ ^23^ ^]^ Besides, suppressing m^6^A modification by ablating the m^6^A writer Mettl3 or the m^6^A reader Ythdc2 in liver both protects against diet induced fatty liver.^[^ ^21^ ^]^ Mettl14‐deficiency induced m^6^A depletion in pancreatic *β* cells induces cell‐cycle arrest and impairs insulin secretion,^[^ ^20^ ^]^ while a recent study showed that disrupting Mettl3 specifically in BAT impaired its postnatal development and thermogenic functions.^[^ ^24^ ^]^ However, the detailed functions of m^6^A modifications in beige adipocytes and how it impacts glucose metabolism and energy homeostasis remain mysterious. Considering the great therapeutic potentials of beige adipocytes in clinic, further investigation on this field is worthwhile.^[^ ^6^, ^7^ ^]^

In the present study, via MeRIP‐sequencing and RNA‐sequencing combined with screens of m^6^A regulators in mice beige fat, we found that m^6^A writer Mettl3 plays a major role in beige fat activation by regulating glycolysis. Subsequent analysis of fat‐specific Mettl3 knockout mice, as well as AAV‐mediated beige fat‐specific Mettl3 knockdown or overexpression mice models revealed that Mettl3 impacts beige fat thermogenic capacity and mice energy expenditure by regulating glycolytic genes, including Hk2, Pfkl, and Pkm, which was mediated by m^6^A reader Igf2bp2. Furthermore, Mettl3 regulates beige preadipocyte proliferation by mediating crosstalk between mature adipocytes and preadipocytes via the glycolytic product lactate. Overall, the present study highlighted a unique role of Mettl3 in beige fat functionality and systemic energy homeostasis.

## 2 Results

### 2.1 Mettl3 Upregulated Glycolytic Genes by Inducing m^6^A Modification in Cold Induced Beige Adipocyte Activation

In order to study the possible role of m^6^A modification in the process of beige adipocyte activation, we set two groups of 2‐month‐old C57BL6/J male mice at either thermoneutral temperature of 30 °C or under cold stimulation at 4 °C for 1 week. Compared to mice at thermoneutrality, 7 days cold exposure led to reduced inguinal white adipose tissue (iWAT) weights in mice, while histological analysis showed largely increased multilocular beige adipocytes with reduced adipocyte sizes, accompanied with dramatically enhanced UCP1 immunohistological staining and increased expressions of brown gene programs (Figure S1A–C, Supporting Information). Overall, these data suggested that cold stimulation induced strong beige fat activation in mice iWAT.

**Figure 1:**
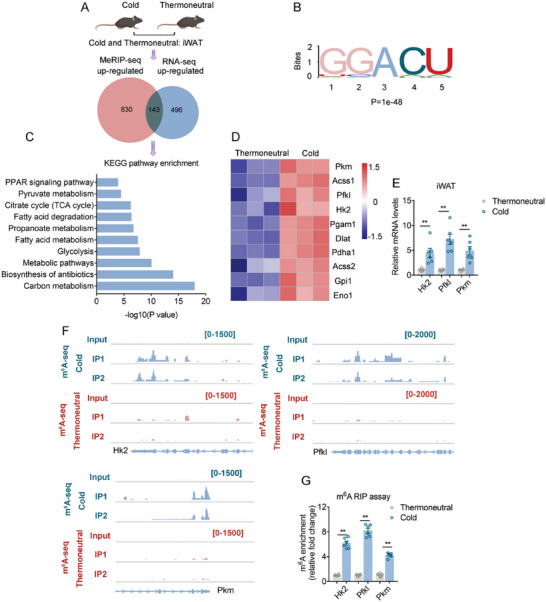
m^6^A modification induced glycolysis activation of beige fat in cold. A) Venn diagram showing overlapped genes with increased m^6^A modification in MeRIP‐seq and up‐regulated mRNA levels in RNA‐seq in iWAT from mice under chronic cold stimulation or thermoneutral condition. B) N6‐methyladenosine (m^6^A) peaks were identified by HOMER. C) Representative KEGG analysis on overlapped genes identified in (A). D) Heatmap showing genes enriched in Glycolysis pathway identified in (C). E) Relative mRNA levels of glycolytic key genes including Hk2, Pfkl, and Pkm in iWAT from mice under thermoneutral or cold by qRT‐PCR (*n* = 6). F) m^6^A peaks in glycolytic key genes including Hk2, Pfkl, and Pkm. G) m^6^A enrichment of Hk2, Pfkl, and Pkm in iWAT from mice under chronic cold stimulation or thermoneutral condition by MeRIP‐qPCR (*n* = 6). Data are shown as mean ± SEM. Statistical significance was analyzed by unpaired Student's *t*‐test (E, G). *p < 0.05, **p < 0.01.

Interestingly, cold stimulation induced higher whole m^6^A modification level in beige fat compared to thermoneutrality, suggesting m^6^A is involved in the regulation of beige fat (Figure S1D, Supporting Information). Then, to identify events vital to beige fat activation, we performed MeRIP‐seq and RNA‐seq using mice iWAT and focused on differentially‐expressed genes characterized significant upregulation. Overlapping of sequencing data from MeRIP‐seq and RNA‐seq identified 143 mRNAs featured concomitant elevation in m^6^A modification levels and mRNA expression levels, suggesting they might be key genes for beige fat activation that are regulated by m^6^A modification (**Figure** **1**A,B). Further KEGG pathway analysis on these genes enriched pathways mainly involved in glucose metabolism, including carbon metabolism as top enriched pathway, as well as pathways involving glycolysis, pyruvate metabolism, and TCA cycle (Figure 1C). Moreover, a close look at the gene list of the top enriched carbon metabolism pathway revealed that the majority of genes are annotated as glycolysis genes (Table S1, Supporting Information), including the rate limiting genes of the glycolytic pathway Hk2, Pfkl, and Pkm,^[^ ^25^ ^]^ which were further confirmed by qPCR analysis (Figure 1D,E). Moreover, the m^6^A modification levels of these genes were also enhanced (Figure 1F,G). These results suggested that glycolysis may play a critical role in the metabolic acclimation of iWAT during chronic cold stimulation, which characterized increased mRNA abundance of glycolytic genes possibly through enhanced m^6^A modification. On the other hand, KEGG pathway analysis on down‐regulated gene from overlap of MeRIP‐seq and RNA‐seq enriched pathways mainly involved in cancer progression (Figure S1E, Supporting Information), which is consistent with the notion that beige fat activation through cold exposure played an active role in combating cancer.^[^ ^26^ ^]^

**Figure 2:**
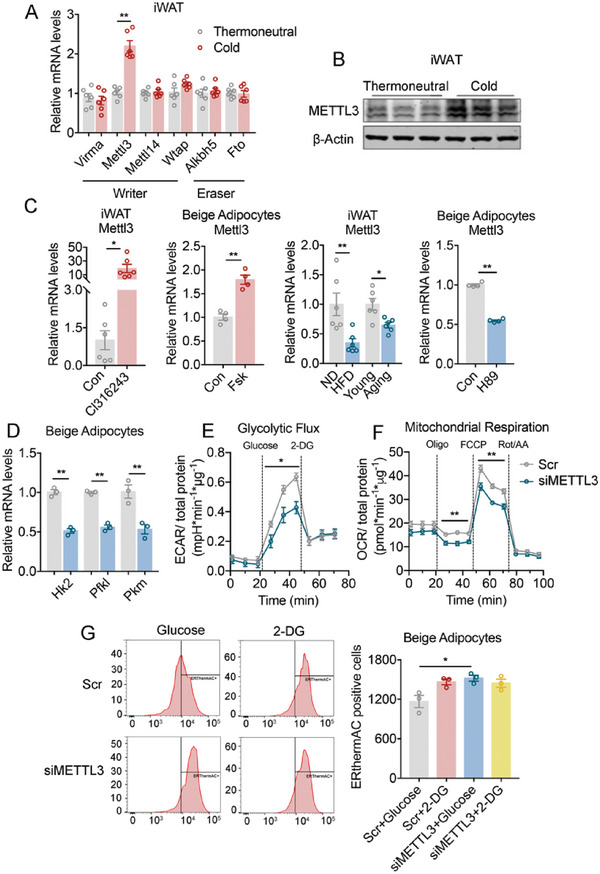
Mettl3 levels are associated with metabolic status and Mettl3 impacts thermogenesis through its regulation on glycolysis in beige adipocytes. A) Relative mRNA levels of major methyltransferases (writer) and demethylases (eraser) in iWAT of mice under thermoneutral or cold (*n* = 6). B) Protein level of METTL3 determined by western blot in iWAT of mice under thermoneutral or cold. C) Relative mRNA level of Mettl3 in iWAT of mice treated with *β*‐adrenergic receptor agonist Cl316243, in iWAT of HFD mice or aging mice versus their controls (*n* = 6), and in beige adipocytes treated by Forskolin (Fsk) or H89 versus their controls (*n* = 4). D) Relative mRNA levels of glycolytic genes in beige adipocytes treated with scramble (Scr) or siMettl3 (*n* = 3). E) Extracellular acidification rate (ECAR) in beige adipocytes treated with scramble (Scr) or siMettl3 (*n* = 5). F) Oxygen consumption rate (OCR) in beige adipocytes treated with scramble (Scr) or siMettl3 (*n* = 3). G) FACS analysis and quantification of ERthermAC in beige adipocytes treated with scramble (Scr) or siMettl3, then administrated with glucose or 2‐DG (*n* = 3). Data are shown as mean ± SEM. Statistical significance was analyzed by unpaired Student's *t*‐test (A, C, D, G) or two‐way ANOVA followed with Bonferroni's multiple comparisons test (E, F). *p < 0.05, **p < 0.01.

Next, to find out the factors responsible for the elevated m^6^A modifications on glycolytic genes during cold stimulation, we screened expression levels of a panel of m^6^A writers and erasers in mice kept under thermoneutral or cold environment, which revealed a specific induction in m^6^A writer Mettl3 in iWAT in both mRNA and protein levels upon cold challenge (**Figure** **2**A,B; Figure S1F, Supporting Information). Furthermore, in both in vivo and in vitro models, Mettl3 levels increased upon treatments that activated beige fat function, that is, CL316243 or forskolin administration, while its levels decreased under manipulations that suppressed beige fat activation, that is, high fat diet (HFD), aging, or *β*‐adrenergic receptor blocker H89 (Figure 2C), indicating the involvement of Mettl3 in beige fat activation.

Considering beige adipocytes activation altered mainly glycolytic gene transcription and m^6^A modulation, we then tested whether Mettl3 regulates glycolytic gene programs. Indeed, we found that Mettl3 knockdown (siMettl3) in differentiated beige adipocytes significantly suppressed critical glycolytic genes Hk2, Pfkl, and Pkm, accompanied with reduced extracellular acidification rate (ECAR) and oxygen consumption rate (OCR), indicators for glycolytic flux to lactate and mitochondrial respiration, respectively (Figure 2D–F). These results showed that Mettl3 loss in beige adipocytes impaired both glycolysis and thermogenesis. To determine whether Mettl3‐promoted glycolysis is required for the enhancement of thermogenesis in beige fat, we treated cells with 2‐deoxyglucose (2‐DG) to inhibit glycolysis, and then monitor temperature changes in live beige adipocytes with a small molecule thermosensitive fluorescent dye, ERthermAC, which signals increased temperature with decreased fluorescent intensity.^[^ ^5^, ^27^ ^]^ Consistent with previous reports that beige adipocyte glycolysis contributes to thermogenesis,^[^ ^9^, ^28^ ^]^ glucose‐treated beige adipocytes had increased temperature (decreased ERthermAC fluorescent intensity) compared with 2‐DG treated group (Figure 2G). Of note, this effect of glycolysis was significantly blunted upon Mettl3 deficiency, while Mettl3 knockdown in 2‐DG‐treated beige adipocytes did not further suppress their thermogenic capability (Figure 2G), suggesting Mettl3 impacts beige adipocyte thermogenesis through its regulation on glycolysis. Taken together, these results offered a unique correlation between Mettl3 level and the expression of glycolysis gene program in beige adipocyte, possibly through altering m^6^A abundance on mRNA of glycolytic genes.

### 2.2 Genetic Ablation of Mettl3 in Adipose Tissues Led to Reduced Glycolytic and Thermogenic Capability

**Figure 3:**
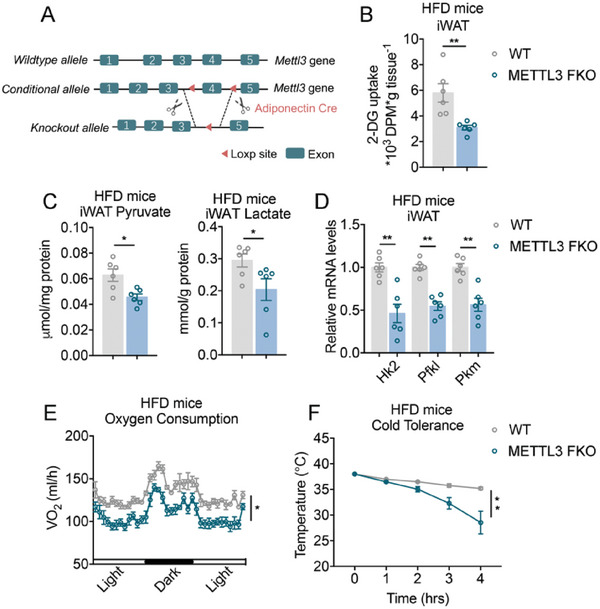
Genetic ablation of Mettl3 in adipose tissues led to reduced glycolytic and thermogenic capability. A) Construction strategy of Adiponectin‐Cre Mettl3^loxp/loxp^ mice for Mettl3‐specific knockout in fat (Mettl3 FKO). B) The levels of 2‐DG uptake in iWAT of WT and Mettl3 FKO mice on HFD (*n* = 6). C) The levels of pyruvate and lactate in iWAT of WT and Mettl3 FKO mice on HFD (*n* = 6). D) Relative mRNA levels of glycolytic genes in iWAT of WT and Mettl3 FKO on HFD (*n* = 6). E,F) Analysis of metabolic parameters of WT and Mettl3 FKO mice on HFD, including oxygen consumption (E) and (F) cold tolerance (*n* = 6) (F). Data are shown as mean ± SEM. Statistical significance was analyzed by unpaired Student's *t*‐test (B–D), two‐way ANOVA followed with Bonferroni's multiple comparisons test (F) or ANCOVA with body weight as covariant (E). *p < 0.05, **p < 0.01.

Previous study reported that brown fat‐specific Mettl3 knockout blunted brown fat development and promoted diet induced obesity and systemic insulin resistance in mice.^[^ ^24^ ^]^ To comprehensively evaluate the in vivo role of Mettl3 specifically on fat biology, we crossed Adiponectin‐Cre with Mettl3‐Loxp mice to obtain a Mettl3 fat‐specific genetic ablation mice model (Mettl3 FKO) (**Figure** **3**A; Figure S2A, Supporting Information). Comprehensive characterizations of the metabolic performances of mice on normal chow diet (NCD) showed that WT and Mettl3 FKO mice have similar body weights and body mass compositions, comparable white fat tissue weights (iWAT and epididymal white adipose tissue), while BAT (brown adipose tissue) of FKO mice is lager, as consistent with previous report^[^ ^24^ ^]^ (Figure S2B,C, Supporting Information). Metabolic analysis showed no changes in oxygen consumption or insulin sensitivity between WT and Mettl3 FKO mice (Figure S2D,E, Supporting Information). Although glycolytic gene expression in iWAT of Mettl3 FKO mice under NCD exhibited mild but significant decrease compared to WT (Figure S2F, Supporting Information), basal glycolysis in iWAT of WT and FKO mice were similar as shown by the comparable pyruvate and lactate levels in iWAT, possibly due to a lack of metabolic stress under normal diet.

Next, we analyzed the metabolic performances of WT and Mettl3 FKO mice under metabolic challenge by subjecting them on HFD. Consistent with the in vitro results that Mettl3 regulate glycolysis in beige adipocytes, Mettl3 knockout in adipose tissue suppressed glucose uptake, the levels of pyruvate and lactate in iWAT, accompanied with reduced glycolytic, Glut4 and thermogenic genes (Figure 3B–D; Figure S2G, Supporting Information), suggesting that genetic knockout of Mettl3 reduced glucose uptake and glycolytic flux in iWAT. Interestingly, we also found that deletion of Mettl3 significantly impaired key glycolytic genes in BAT, in addition to previously reported reduced thermogenic genes (Figure S2H, Supporting Information), suggesting the regulation of Mettl3 on glycolysis may be a common mechanism in thermogenic fat. Besides, Mettl3 deficiency resulted in decreased thermogenic capability and oxygen consumption, without changes in activity or food intake (Figure 3E,F; Figure S2I, Supporting Information).

In order to specifically examine the contribution of Mettl3 to beige fat functionality and systematic metabolic control, we have surgically removed BAT of WT and Mettl3 FKO mice to exclude the influence of BAT on metabolism. Compared to BAT‐removal WT mice, we found that BAT‐removal Mettl3 FKO mice under HFD still exhibited decreased levels of pyruvate and lactate in iWAT, diminished iWAT glycolytic gene expression, as well as reduced thermogenic capability and oxygen consumption (Figure S2J,K, Supporting Information). These results suggested that Mettl3 deficiency in beige fat had major contribution to impaired glycolytic and thermogenic capacity in mice.

### 2.3 Genetic Ablation of Mettl3 Reduces Proliferation of Preadipocytes and iWAT Mass

**Figure 4:**
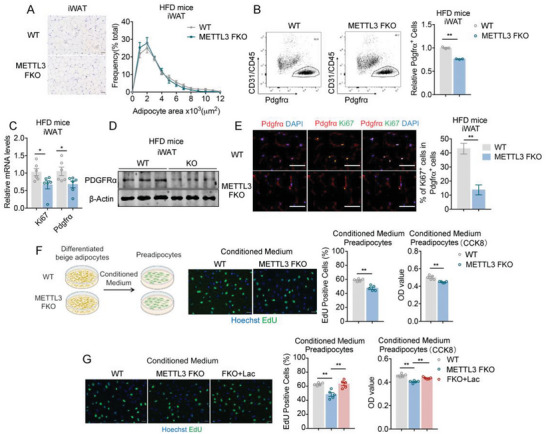
Genetic ablation of Mettl3 reduces proliferation of preadipocytes and iWAT mass. A) Representative images of H&E staining of iWAT (left) and the frequency distribution of adipocytes area in iWAT (right) of WT and Mettl3 FKO mice on HFD (*n* = 6). B) Representative cell sorting of CD31^−^ CD45^−^ Pdgfr*α* ^+^ cells from iWAT SVF of HFD‐fed WT and Mettl3 FKO mice by FACS (left) and quantification of the relative CD31^−^ CD45^−^ Pdgfr*α* ^+^ in SVF cell from iWAT (right) (*n* = 3). C) Relative mRNA levels of Ki67 and Pdgfr*α* in iWAT of WT and Mettl3 FKO mice on HFD (*n* = 6). D) Protein level of PDGFR*α* determined by western blot in iWAT of WT and Mettl3 FKO mice on HFD. E) Representative images of Pdgfr*α* and Ki67 staining in iWAT sections of WT and Mettl3 FKO mice on HFD (left) and quantification of the percentage of Pdgfr*α* ^+^ Ki67^+^ cells in Pdgfr*α* ^+^ cells in iWAT (right) (*n* = 6). F) EdU staining and Cell Counting Kit‐8 (CCK8) assay of preadipocytes treated with conditioned medium from primary WT or Mettl3 FKO adipocytes (*n* = 5). G) EdU staining and CCK8 assay of preadipocytes treated with conditioned medium from primary adipocytes of WT, Mettl3 FKO, or Mettl3 FKO supplemented with lactate (*n* = 5). Data are shown as mean ± SEM. Statistical significance was analyzed by unpaired Student's *t*‐test (B, C, E–G) or two‐way ANOVA followed with Bonferroni's multiple comparisons test (A). *p < 0.05, **p < 0.01. Scale bars, 50 µm.

Surprisingly, although glycolysis and energy metabolism were reduced in FKO mice, we found that genetic Mettl3 knockout in fat resulted in significantly reduced body weight and fat mass in HFD mice as compared to controls, with or without the presence of BAT (Figure S2L,M, Supporting Information). Detailed analysis showed that although BAT was larger in Mettl3 FKO mice as previously reported,^[^ ^24^ ^]^ subcutaneous and epididymal fat were much smaller (Figure S2L, Supporting Information). Interestingly, a closer look at the iWAT adipocyte sizes showed no obvious differences between WT and Mettl3 FKO mice (**Figure** **4**A). Thus, we looked at whether Mettl3 deficiency would impact preadipocytes in iWAT. Flow cytometry analysis revealed reduced CD31^−^CD45^−^Pdgfr*α* ^+^ preadipocytes in iWAT of Mettl3 FKO mice (Figure 4B). Consistently, we also found reduced levels of proliferative marker Ki67 and the adipocyte progenitor marker Pdgfr*α*, as well as decreased proliferative progenitor cell numbers in iWAT of FKO mice compared to WT mice (Figure 4C–E). Importantly, we found that manipulating Mettl3 levels of preadipocytes (siMettl3 or Mettl3) did not change their proliferation compared to controls, indicating that adipocytes–preadipocytes crosstalk may underline this phenomenon (Figure S3A,B, Supporting Information).

It has been reported that increased output of lactate, the glycolytic metabolic product, from muscle due to aberrant Ca^2+^ release in a malignant hyperthermia susceptibility mouse model increased preadipocyte proliferation in brown fat.^[^ ^29^ ^]^ Considering that Mettl3 FKO mice featured decreased lactate levels in iWAT under HFD due to reduced glycolysis, we hypothesized that the smaller white fat mass in Mettl3 FKO HFD mice might result from impaired glycolysis and the subsequent lactate production, which inhibit progenitor hyperplasia. Indeed, lactate treatment significantly enhanced beige preadipocytes proliferation (Figure S3C, Supporting Information). Importantly, we collected conditioned medium from differentiated primary beige adipocytes from iWAT of WT or Mettl3 FKO mice to treat primary preadipocytes, which revealed that medium from FKO beige adipocytes significantly decreased the number of proliferating (EdU positive) preadipocytes and growth rates of preadipocyte (Figure 4F), suggesting Mettl3 in mature beige adipocytes regulates the proliferation of preadipocytes. Moreover, lactate administration rescued the reduced proliferation of beige preadipocytes treated with conditioned medium from FKO beige adipocytes (Figure 4G). We also performed rescue experiment by overexpressing key glycolytic genes Hk2, Pfkl, and Pkm via lentiviral delivery into mature beige adipocytes of Mettl3 FKO mice. We collected conditioned medium from these cells, as well as conditioned medium from mature beige adipocytes of WT and Mettl3 FKO mice, to treat preadipocytes and evaluated preadipocyte proliferation. The results showed that, compared to WT, conditioned medium from Mettl3 FKO beige adipocytes inhibited preadipocytes proliferation, which were reversed, at least partially, upon glycolytic genes overexpression in Mettl3 FKO cells (Figure S3D, Supporting Information). Besides, we analyzed the markers of different types of mature adipocytes differentiated from Mettl3‐knockdown preadipocytes or in lactate‐treated preadipocytes, indicating that changes in m^6^A modification upon Mettl3 knockdown or local lactate supplementation has no effect on differentiation direction of beige preadipocytes (Figure S3E, Supporting Information). These data indicate that Mettl3 mediated glycolytic production of lactate in mature beige adipocytes may promote preadipocytes proliferation.

Overall, these data suggested that although Mettl3 knockout suppressed glycolysis and thermogenesis in iWAT, different than the previous reported BAT‐specific Mettl3 knockout mice,^[^ ^24^ ^]^ Mettl3 FKO mice were resistant to obesity under energy challenge, at least partially due to reduced proliferation of preadipocytes in iWAT by glycolytic product lactate mediated crosstalk between mature adipocytes and preadipocytes. Of note, we did not observe increased lipid deposition in liver in FKO mice compared to WT (Figure S3F, Supporting Information). Considering that both groups of mice had similar food intake and activity, while FKO mice had reduced lipid accumulation in white fat depots, it is possible that the extra energy may be ectopically stored inside the dysfunctional and enlarged FKO BAT. In addition, we have examined the fecal Triglyceride (TG) content and found that the feces of Mettl3 FKO mice showed increased fecal TG. Besides, small intestinal TG quantification analysis indicated that Mettl3 FKO mice had decreased small intestinal TG contents, suggesting that small intestine of Mettl3 FKO mice may inhibit intestinal lipid absorption (Figure S3G, Supporting Information). Thus, the intestinal lipid absorption in Mettl3 FKO mice may be decreased, whereas energy efflux is increased via feces excretion, which may contribute to the reduced body weight and fat mass in these mice.

### 2.4 Mettl3 Deficiency in iWAT Reduces Glucose Metabolism, Thermogenic Capacity, and Fat Weights in Mice

**Figure 5:**
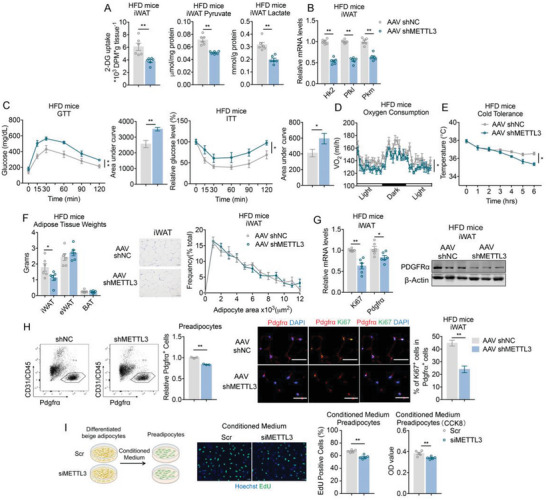
Mettl3 deficiency in iWAT reduces glucose metabolism, thermogenic capacity, and fat weights in mice. A) The levels of 2‐DG uptake, pyruvate and lactate in iWAT of mice with AAV mediated NC (AAV shNC) or Mettl3 knockdown (AAV shMettl3) in inguinal fats and maintained on HFD for 6weeks (*n* = 6). B) Relative mRNA levels of glycolytic genes in iWAT of AAV shNC and AAV shMettl3 mice on HFD for 6 weeks (*n* = 6). C–E) Analysis of metabolic parameters of AAV shNC and AAV shMettl3 mice on HFD for 6 weeks, including GTT (C) and ITT (*n* = 6); oxygen consumption (*n* = 5) (D), and cold tolerance (*n* = 6) (E). F) Adipose tissues weights (left), representative images of H&E staining of iWAT (middle) and the frequency distribution of adipocytes area in iWAT (right) of AAV shNC and AAV shMettl3 mice on HFD for 6 weeks (*n* = 6). G) Relative mRNA levels of Ki67 and Pdgfr*α* (*n* = 6) and protein level of PDGFR*α* determined by western blot in iWAT of AAV shNC and AAV shMettl3 mice on HFD for 6 weeks. H) Representative cell sorting of CD31^−^ CD45^−^ Pdgfr*α* ^+^ cells from iWAT SVF of HFD‐fed AAV shNC and AAV shMettl3 mice by FACS (left) (*n* = 3) and representative images of Pdgfr*α* and Ki67 staining in iWAT sections of AAV shNC and AAV shMettl3 mice on HFD (right) (*n* = 6). I) EdU staining and CCK8 assay of preadipocytes treated with conditioned medium from differentiated beige adipocytes with or without Mettl3 knockdown (*n* = 5). Data are shown as mean ± SEM. Statistical significance was analyzed by unpaired Student's *t*‐test (A,B,F–I), two‐way ANOVA followed with Bonferroni's multiple comparisons test (C,E,F), or ANCOVA with body weight as covariant (D). *p < 0.05, **p < 0.01. Scale bars, 50 µm.

Since Mettl3 FKO mice had Mettl3 deficiency in both iWAT and epididymal white adipose tissue (eWAT), to decipher the role of Mettl3 on beige fat, we specifically examined the in vivo functions of Mettl3 on glycolysis and thermogenesis in mice beige fat by injecting AAV‐mediated control (AAV shNC) or Mettl3 shRNA (AAV shMettl3) bilaterally in subcutaneous fat of mice under HFD to achieve efficient and selective knockdown of Mettl3 in iWAT (Figure S4A, Supporting Information). Consistent with what were observed in genetic FKO mice, Mettl3 knockdown in mice iWAT significantly suppressed glucose uptake and decreased the levels of glycolytic metabolites pyruvate and lactate (**Figure** **5**A), while simultaneously reduced mRNA abundance of glycolytic, Glut4, and thermogenic genes (Figure 5B; Figure S4B, Supporting Information), suggesting that Mettl3 is required for glycolysis in iWAT. Moreover, compared to control mice, AAVshMettl3 group exhibited significantly impaired insulin sensitivity and glucose tolerance in glucose and insulin tolerance tests (GTT and ITT) (Figure 5C), indicating specific Mettl3 deficiency in beige fat had major impacts on glucose utilization and glycolysis. In addition, AAV‐shMettl3 HFD mice featured decreased energy expenditure and thermogenic capability, as evident by the decreased oxygen consumption, as well as defects in defending their core temperature during a cold challenge compared to control mice (Figure 5D,E).

Considering AAV‐mediated gene ablation is a relative acute process compared to the genetic ablation model, it is not surprising that body weights and fat mass were comparable between two groups (Figure S4C, Supporting Information), while a close look at fat depots revealed that, similar to Mettl3 FKO mice, iWAT weights were reduced in AAV‐shMettl3 HFD mice, while iWAT adipocyte sizes appeared to be similar between the two groups (Figure 5F). We then investigated whether the reduced iWAT weights were also due to the regulation of Mettl3 on preadipocytes proliferation. At the molecular level, we found reduced levels of Pdgfr*α* and Ki67 in iWAT upon Mettl3 deficiency (Figure 5G). Besides, the percentage of CD31^−^CD45^−^Pdgfr*α* ^+^ labeling preadipocytes in FACS analysis, as well as the ratio of proliferating preadipocytes marked by Ki67 and PDGFR*α* immunofluorescent double staining were decreased in AAV‐shMettl3 mice iWAT (Figure 5H). In addition, when treated with conditioned medium from differentiated beige adipocytes of Mettl3 knockdown (siMettl3), preadipocytes displayed reduced proliferation compared to control (Scr) (Figure 5I). Overall, these phenotypes recapitulated the genetic ablation of Mettl3 in iWAT and highlighted the vital role of Mettl3 in glucose metabolism, thermogenic capacity and beige fat biology.

### 2.5 Mettl3 Overexpression in iWAT Increased Glucose Metabolism, Thermogenic Capacity, and Fat Weights in Mice

**Figure 6:**
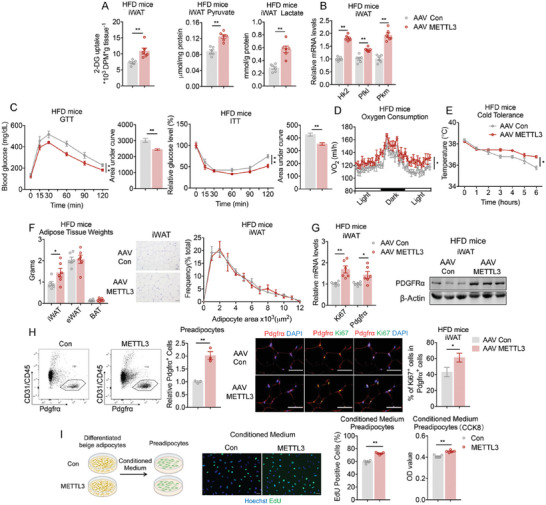
Mettl3 overexpression in iWAT increased glucose metabolism, thermogenic capacity and fat weights in mice. A) The levels of 2‐DG uptake, pyruvate and lactate in iWAT of mice with AAV mediated Control (AAV Con) or Mettl3 overexpression (AAV Mettl3) in inguinal fats on HFD for 10 weeks (*n* = 6). B) Relative mRNA levels of glycolytic genes in iWAT of AAV Con and AAV Mettl3 mice on HFD for 10 weeks (*n* = 6). C–E) Analysis of metabolic parameters of AAV Con and AAV Mettl3 mice, including GTT (C) and ITT (*n* = 6); D) Oxygen consumption (*n* = 5) and E) cold tolerance (*n* = 6). F) Adipose tissues weights (left), representative images of H&E staining of iWAT (middle) and the frequency distribution of adipocytes area in iWAT (right) of AAV Con and AAV Mettl3 mice on HFD for 10 weeks (*n* = 6). G) Relative mRNA levels of Ki67 and Pdgfr*α* (*n* = 6) and protein level of PDGFR*α* determined by western blot in iWAT of AAV Con and AAV Mettl3 mice on HFD for 10 weeks. H) Representative cell sorting of CD31^−^ CD45^−^ Pdgfr*α* ^+^ cells from iWAT SVF of HFD‐fed AAV Con and AAV Mettl3 mice by FACS (left) (*n* = 3) and representative images of Pdgfr*α* and Ki67 staining in iWAT sections of AAV Con and AAV Mettl3 mice on HFD (right) (*n* = 6). I) EdU staining and CCK8 assay of preadipocytes treated with conditioned medium from differentiated beige adipocytes with or without Mettl3 overexpression (*n* = 5). Data are shown as mean ± SEM. Statistical significance was analyzed by unpaired Student's *t*‐test (A,B,F–I), two‐way ANOVA followed with Bonferroni's multiple comparisons test (C,E,F) or ANCOVA with body weight as covariant (D). *p < 0.05, **p < 0.01. Scale bars, 50 µm.

To further demonstrate the role of Mettl3 in beige fat biology, we subsequently delivered Con (AAV Con) or Mettl3 (AAV Mettl3) bilaterally in iWAT of HFD mice to achieve specific Mettl3 overexpression in iWAT (Figure S4D, Supporting Information). Notably, Mettl3 overexpression in mice iWAT showed opposite phenotypes as compared to the previous Mettl3 knockdown mice models, and characterized enhanced glucose uptake, elevated pyruvate and lactate levels (**Figure** **6**A), increased glycolytic, Glut4 and thermogenic genes expression in iWAT (Figure 6B; Figure S4E, Supporting Information), improved insulin sensitivity and glucose metabolism (Figure 6C), along with increased energy expenditure and cold tolerance capacity (Figure 6D,E) in AAV Mettl3 group versus control group. Furthermore, iWAT weights of AAV Mettl3 HFD mice were increased, without changes in iWAT adipocyte sizes, whole body weights or fat mass (Figure 6F; Figure S4F, Supporting Information).

In parallel, we found that Ki67 and Pdgfr*α* levels were significantly increased in iWAT upon Mettl3 overexpression in AAV Mettl3 group (Figure 6G), along with increased percentage of preadipocytes and proliferating preadipocytes in their iWAT compared to controls (Figure 6H). Moreover, as opposed to Mettl3 knockdown, conditioned medium from differentiated beige adipocytes of Mettl3 overexpression promoted preadipocytes proliferation (Figure 6I).

Overall, these data suggested that converse to Mettl3 deficiency in iWAT, Mettl3 overexpression promotes glycolysis in beige fat and improves systemic glucose metabolism and thermogenic capacity, as well as enhancing beige preadipocytes proliferation.

### 2.6 Mettl3 and m^6^A Reader Igf2bp2 Regulate mRNA Stability of Glycolytic Genes in Beige Adipocytes

**Figure 7:**
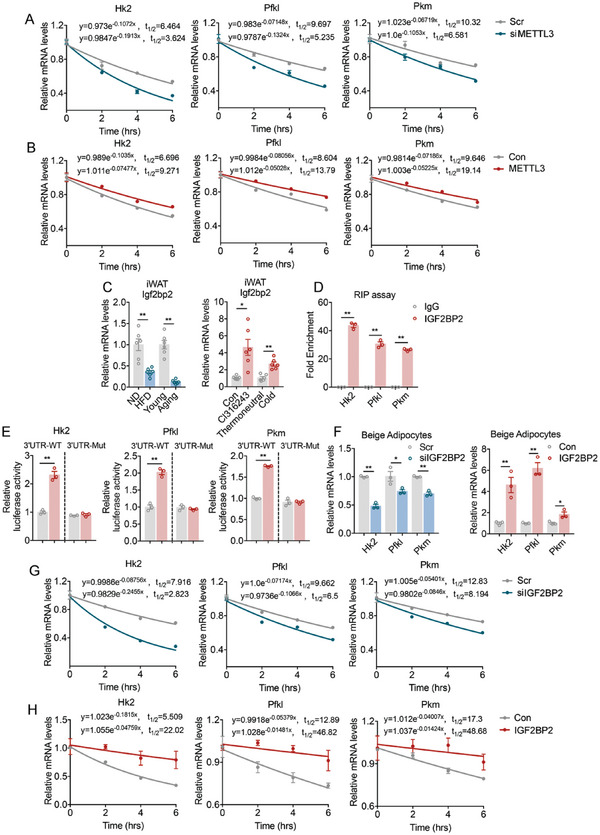
Mettl3 and Igf2bp2 promotes mRNA stability of glycolytic genes in beige adipocytes. A) mRNA levels of glycolytic genes in scramble (Scr) or Mettl3 knockdown (siMettl3) beige adipocytes upon transcriptional inhibition with actinomycin D at indicated time (*n* = 3). B) mRNA levels of glycolytic genes in ADV mediated Con or Mettl3 overexpression (Mettl3) beige adipocytes upon transcriptional inhibition with actinomycin D at indicated time (*n* = 3). C) mRNA level of Igf2bp2 in iWAT of HFD‐fed or aging mice versus their controls (left, *n* = 6); in iWAT of CL316243 or cold treated mice versus their controls (right, *n* = 6). D) RIP assay assessing Igf2bp2 binding on 3′UTR of Hk2, Pfkl, and Pkm in differentiated beige adipocytes (*n* = 3). E) Relative luciferase activity of glycolytic genes WT‐3′UTR luc‐construct or m^6^A site mutant‐3′UTR (A‐to‐T mutation) luc‐construct after cotransfection with Igf2bp2 in HEK293T cells (*n* = 3). F) Relative mRNA levels of glycolytic genes in beige adipocytes treated with scramble (Scr) or siIgf2bp2 (left, *n* = 3), or ADV mediated Con or Igf2bp2 overexpression (Igf2bp2) (right, *n* = 3). G) mRNA levels of glycolytic genes in scramble (Scr) or Igf2bp2 knockdown (siIgf2bp2) beige adipocytes upon transcriptional inhibition with actinomycin D at indicated time (*n* = 3). H) mRNA levels of glycolytic genes in ADV mediated Con or Igf2bp2 overexpression (Igf2bp2) beige adipocytes upon transcriptional inhibition with actinomycin D at indicated time (*n* = 3). Data are shown as mean ± SEM. Statistical significance was analyzed by unpaired Student's *t*‐test. *p < 0.05, **p < 0.01. Scale bars, 50 µm.

We further elucidated how Mettl3 regulates the expression of glycolysis genes. As a m^6^A writer, Mettl3 functions by enhancing m^6^A modification on mRNAs, which has been shown to be important for mRNA stability.^[^ ^30^, ^31^ ^]^ We thus tested whether Mettl3 regulates glycolytic gene programs via mRNA stability by performing RNA lifetime profiling after Mettl3 knockdown or overexpression in mature beige adipocytes. The RNA stability curves showed that knockdown of Mettl3 reduced half‐lives of Hk2, Pfkl, and Pkm mRNA in mature beige adipocytes, while overexpression of Mettl3 significantly increased their mRNA stability (**Figure** **7**A,B). Thus, Mettl3 induced upregulation of Hk2, Pfkl, and Pkm levels at least in part by increasing the stability of their mRNA transcripts.

We then intended to look for the effector that mediates Mettl3's regulation on glycolytic mRNA. Insulin‐like growth factor 2 mRNA binding protein 2 (Igf2bp2) is a well‐established m^6^A reader in mammalian cells. It has been shown to promote glycolysis by binding to the 3′ untranslated region (3′UTR) of Hk2 mRNA and stabilizing Hk2 mRNA in cancers.^[^ ^31^, ^32^ ^]^ We examined whether Igf2bp2 is involved in beige fat glycolytic functionality by recognizing mRNAs modified by Mettl3. Of note, we found that Igf2bp2 mRNA levels are tightly associated with beige fat function in a similar fashion as Mettl3, since Igf2bp2 was downregulated in iWAT in scenarios that suppressing beige fat function, that is, in HFD mice or aging mice, while conversely, Igf2bp2 showed increased expressions in iWAT in conditions that promoting white fat browning, including CL316243 treatment and cold stimulation (Figure 7C). Using RIP‐qPCR analysis, we revealed the existence of specific Igf2bp2‐binding site on 3′UTR of Hk2, Pfkl, and Pkm transcripts (Figure 7D). We then used SRAMP software to identify a high‐confidence m^6^A site in the 3′UTR region of these mRNAs.^[^ ^33^ ^]^ Based on this, we performed luciferase reporter assays using luciferase reporters containing 3′UTR region of Hk2, Pfkl, and Pkm (3′UTR‐WT) or mutated 3′UTR region with the m6A modification site GGm^6^ACT mutated to GGTCT (3′UTR‐MUT) (Figure S5A, Supporting Information). As expected, Igf2bp2 increased the activity of 3′UTR‐WT of Hk2, Pfkl, and Pkm, while these effects were abolished in 3′UTR‐MUT (Figure 7E), suggesting that Igf2bp2 recognizes and directly binds to 3′UTR of glycolytic genes. Echoing the results of luciferase analysis, Igf2bp2 knockdown in beige adipocytes resulted in reduced expression and mRNA stability of glycolytic genes, while Igf2bp2 overexpression enhanced mRNA levels and stability of glycolytic genes (Figure 7F–H), suggesting Igf2bp2 may function through enhancing mRNA stability of glycolytic gene program. We also examined the mRNA levels of various other m6A reader in mice under cold and thermoneutral environment, which showed that four members of the YTH domain family (Ythdc2, Ythdf1, Ythdf2, and Ythdf3) were upregulated during cold stimulation. We then depleted these readers individually in beige adipocytes to see their impact on glycolysis and found that none of the four YTH domain family readers can affect key glycolytic genes Hk2, Pfkl, and Pkm simultaneously as Igf2bp2 did (Figure S5B, Supporting Information). Thus, Igf2bp2 is the only m^6^A reader that could inhibit key glycolytic gene expression levels in response to cold.

In addition, we found that preadipocytes proliferation was reduced upon treatment with conditioned medium from siIgf2bp2 beige adipocytes, while proliferation was promoted in response to conditioned medium from Igf2bp2‐overexpressing beige adipocytes, suggested that Igf2bp2 in mature beige adipocytes also regulate preadipocyte proliferation in a similar way to Mettl3 (Figure S5C,D, Supporting Information).

Overall, these results support that Mettl3‐mediated m^6^A modification increases mRNA levels of glycolytic genes via Igf2bp2‐dependent regulation on mRNA stability.

## 3 Discussion

As one of the most prevalent, abundant, and conserved modifications in eukaryotic mRNAs, m^6^A modification has been reported to play an essential role in various physiological and pathological conditions.^[^ ^18^, ^19^ ^]^ Of note, m^6^A modification has been shown to be closely associated with numerous metabolic diseases including T2D, fatty liver, and obesity.^[^ ^20^, ^21^, ^22^ ^]^ Adipose tissues play key roles in metabolic diseases, yet the functions and regulatory mechanisms of m^6^A modification in adipose tissues have not been extensively studied. In this study, we reported that the m^6^A writer RNA methyltransferase Mettl3 enhances glycolytic gene mRNA stability via a Igf2bp2‐dependent manner, thus regulates beige fat activation by promoting glycolysis and thermogenesis of beige adipocytes, as well as induces beige preadipocyte proliferation via lactate‐mediated intracellular crosstalk.

In the present study, via MeRIP‐seq and RNA‐seq, we found that the changes in glycolysis are top regulated pathway in iWAT from cold‐stimulated mice, indicating a critical role of glycolysis in beige fat activation. Subsequent screening identified Mettl3 as a major functioning m^6^A writer in beige fat activation. Using a Ucp1‐Cre mediated Mettl3 knockout mice model, Mettl3 was recently reported to regulate m^6^A modifications and expressions of brown‐specific transcripts including Prdm16, Pparg, Ucp1 in BAT, which impacted postnatal BAT development and maturation, in turn influenced BAT‐mediated thermogenesis.^[^ ^24^ ^]^ The phenotypes observed in Ucp1‐Cre mice model may mostly highlight Mettl3's function in brown adipocyte, as it has been reported that Ucp1‐Cre mediated knockout is not efficient in depleting target genes in beige adipocyte, especially in mice without thermogenic challenge.^[^ ^34^, ^35^ ^]^ Considering beige fat's indispensable roles in thermogenesis, we used Adiponectin‐Cre mediated fat‐specific Mettl3 knockout mice (Mettl3 FKO) accompanied with iWAT‐specific Mettl3 manipulation mice models to study Mettl3's regulation on beige adipocytes.^[^ ^36^, ^37^ ^]^ As expected, we also found that Mettl3 FKO mice displayed whitening and enlargement in BAT, which is possibly due to the combined effects of Mettl3 ablation‐caused developmental defects in BAT and the exacerbated lipid accumulation in the dysfunctional BAT due to Mettl3 deficiency‐caused reductions in preadipocyte proliferation and lipid storage capacity in iWAT and eWAT. We also observed impairment in energy expenditure and thermogenesis under HFD in Mettl3 FKO mice, which is at least partially contributed by a specific regulation of Mettl3 in beige fat, since these phenotypes were recapitulated in iWAT‐specific Mettl3 knockout or overexpression mice models, as well as in BAT‐removal Mettl3 knockout mice. Interestingly, recent work demonstrated that adipose‐specific depletion of Mettl3 undermined m^6^A modification of thermogenic mRNAs, including Klf9, thus enhanced their degradation and inhibited WAT beiging.^[^ ^38^ ^]^ Meanwhile, our study demonstrated that Mettl3 and Igf2bp2 directly regulate mRNA stability of glycolytic genes, which impact glycolysis and thermogenesis. Overall, these studies suggested that Mettl3 may regulate glycolytic and thermogenic capacity with multiple mechanisms in adipose tissues. Notably, compared to the role of Mettl3 in BAT development, we demonstrated a unique function of Mettl3 in beige fat thermogenesis via its regulation of glycolytic genes Hk2, Pfkl, and Pkm, as Mettl3 deficiency did not alter white fat development and that iWAT and eWAT of Mettl3 FKO mice appeared normal under basal condition. Moreover, manipulation of Mettl3 levels specifically in iWAT via AAV recapitulated the phenotypical changes of suppressed beige adipocyte glycolysis and reduced thermogenesis observed in Mettl3 FKO mice, indicating a specific function of Mettl3 in beige fat glycolysis compared to its role in BAT maturation. Although function in a similar fashion, beige adipocytes and brown adipocytes are of different developmental origins and feature distinct molecular signature.^[^ ^4^, ^39^ ^]^ Various key regulators have been shown to have different impacts on beige and brown fat function. For example, ablation of Prdm16 or Pgc1*α*, two major thermogenic regulators in fat tissue via Adiponectin‐Cre led to metabolic dysfunctions majorly due to impaired iWAT functionality, instead of changes in BAT,^[^ ^11^, ^12^ ^]^ highlighting the importance of iWAT in energy metabolism. Of note, previous studies on Fto, the first identified m^6^A demethylase, also pointed to different phenotypes in different animal models. Two animal models of Fto fat‐specific knockout mice via Adiponectin‐Cre system were reported, both showed impaired lipolysis and white fat browning, while Fto adipose ablation in one model promoted obesity, and the other model had reduced risk for obesity, suggesting the complexity of m^6^A regulation in obesity^[^ ^40^ ^]^ and highlighted the specificity, timing and efficiency of gene knockout or knockdown in adipose tissues may contribute to different systemic energy homeostasis. These factors may underlie the different regulatory mechanisms of Mettl3 in these two tissues and warrants further investigation.

As an endocrine organ of pleiotropic functions, adipose tissues are classified as white, brown, and beige fat based on their anatomic location and metabolic functions.^[^ ^41^, ^42^ ^]^ m^6^A modification has been shown to regulate adipocyte differentiation, browning and development in different kinds of adipose tissues. For example, m^6^A eraser Fto directly targeted Atg5 and Atg7 transcripts and regulate their expression via a m^6^A‐dependent way. Deletion of Fto enhanced Ythdf2‐mediated Atg5/Atg7 degradation, thus suppressed autophagosome formation, autophagy, and adipogenesis in adipose tissue.^[^ ^43^ ^]^ On the other hand, knockdown of m^6^A writer protein (METTL3, METTL14, or WTAP) in 3T3‐L1 cells results in cell cycle arrest and impaired adipogenesis by suppressing expression of cyclin A2.^[^ ^44^ ^]^ Besides, brown fat‐specific deletion of Mettl3 severely impairs brown adipocyte development and function by decreasing m^6^A modification and expression of Prfm16, Pparg, and Ucp1 transcripts.^[^ ^24^ ^]^ With respect to beige fat and white fat browning, Fto deficiency has been shown to induce Ucp1 expression in white adipocytes,^[^ ^45^ ^]^ whereas our study shows that Mettl3 deficiency in mature beige adipocytes led to suppressed glycolytic capability and thermogenesis, as well as reduced preadipocytes proliferation via glycolytic product lactate, highlighting the significance of m^6^A on systematic energy homeostasis. These results indicate that m^6^A modification plays critical roles in different types of adipose tissues through various mechanisms.

Aerobic glycolysis is one of the most important hallmarks of cancer as cancer cells preferentially utilize glycolysis to meet their high energy needs for supporting tumor growth.^[^ ^46^ ^]^ Previous studies have demonstrated that targeting glycolytic metabolism is a promising strategy for cancer treatment. For example, Li et al demonstrated that Mettl3 was transcriptionally activated by TATA‐binding protein (TBP) in cervical cancer, which induced 5′UTR m^6^A modification of pyruvate dehydrogenase kinase 4 (Pdk4) and promoted Pdk4 recognition by IGF2BP3 and YTHDF1/eEF‐2 complex. This enhanced Pdk4 mRNA stability and translational elongation, respectively, and eventually increased cancer cell glycolysis and tumor growth in a m^6^A dependent manner.^[^ ^47^ ^]^ Interestingly, recent studies have proposed that BAT activation by cold stimulation consumed substantial blood glucose, thus competed for glucose availability to cancer cells and inhibited tumor growth.^[^ ^26^ ^]^ In the present study, we found that Mettl3 is indispensable in cold‐induced beige fat activation. Mettl3 mediated m^6^A modification regulated glycolysis pathway in thermogenic fat and impacted glucose uptake in beige fat by promoting mRNA stability of glycolytic genes, including Hk2, Pfkl, and Pkm, which was mediated by m^6^A reader Igf2bp2. These studies suggested that m^6^A mediated glycolysis plays the common important role of glycolysis regulation in cancer and thermogenic fat, thus presented the potential contribution of m^6^A modification and thermogenic fat glycolysis in combating cancer by energy source competition. Thus, targeting m^6^A modification in thermogenic fat might be a promising strategy for cancer treatment. Future studies on how cold regulates Mettl3 expression and whether m^6^A modification of glycolytic gene mRNA changes their translation are warranted.

Thermogenic fat requires an ample supply of substrates, including both glucose and lipids, to fuel thermogenesis. Although the regulatory mechanisms of lipid utilization in beige and brown fat are extensively studied,^[^ ^48^ ^]^ understandings on the critical contribution of glucose metabolism to thermogenesis are just emerging. For example, Nguyen et al found the NADH oxidase Aifm2 supports robust glycolysis to fuel thermogenesis in BAT, while Winther et al. demonstrated that restriction in glycolysis impairs thermogenic function of brown adipocyte.^[^ ^27^, ^49^ ^]^ Our results offered a post‐transcription mechanism of glycolytic regulation that influence beige fat. Moreover, a new type of glycolytic beige adipocytes is identified in iWAT recently that feature enhanced glucose oxidation and are of different origin and function compared to conventional beige adipocytes.^[^ ^8^ ^]^ It would be worthwhile to investigate whether Mettl3 regulates the formation of these special glycolytic beige adipocytes other than its function on glycolytic genes.

In addition, we found an interesting crosstalk between preadipocytes and mature adipocytes promoted by Mettl3‐induced glycolysis and lactate production. It has been previously reported that lactate promotes brown preadipocytes proliferation.^[^ ^29^ ^]^ Under high fat diet, the proliferation of preadipocytes and their subsequent differentiation into mature adipocytes are required for the production of new adipocytes to maintain adipose tissue mass and metabolic homeostasis to cope with metabolic stresses.^[^ ^50^ ^]^ It seems that Mettl3 induced glycolysis and thermogenesis in mature adipocytes, as well as promoted proliferation in preadipocytes at least partially mediated by lactate, possibly to simultaneously expand beige adipocyte pool and enhance their thermogenic capacity to better cope with energy stress and improve metabolic health.

It is well known that Mettl3 mediated m^6^A modification can be recognized by m^6^A reader proteins which play key roles in regulating gene expression. IGF2BPs tend to maintain mRNA stability.^[^ ^32^ ^]^ We demonstrated that the metabolic effects of Mettl3 are mediated by the m^6^A reader Igf2bp2, which recognizes m^6^A modification on 3′ UTR of glycolytic genes and promotes their mRNA stability in beige fat. Of note, it has been reported that Igf2bp2‐null mice exhibit decreased white adipose tissue mass under HFD due to reduced numbers of Lin‐CD29^+^CD34^+^Sca1^+^ preadipocytes in the stromal‐vascular compartment.^[^ ^51^ ^]^ This is in consistent with our observation in Mettl3 FKO mice and is possibly caused by impaired glycolysis and decreased lactate production. In addition, Igf2bp2 is identified as a Type 2 diabetes‐associated gene and downregulations of Igf2bp2 mRNA level both in visceral or subcutaneous adipose were correlated with insulin resistance in diabetic patients,^[^ ^52^ ^]^ suggesting both Mettl3 and Igf2bp2 may regulate glycolysis in mature white adipocytes and preadipocyte proliferation for glycemic control.

Aside from its role in mRNA stability, Mettl3 also regulate target gene translation by recruiting RBPs such as Ythdf1, Ythdf3, and Igf2bp1/2/3.^[^ ^16^, ^53^, ^54^ ^]^ For example, a recent study reported that Mettl3 enhanced translation elongation of Pdk4 via YTHDF1/eEF‐2 complex and promote glycolysis in cancer cells.^[^ ^47^ ^]^ In the present study, we found that Mettl3 depletion in beige fat blunted the glycolytic gene mRNA level. Thus, we focused on the role of Mettl3 in regulation of mRNA stability. Further studies are warranted to decipher the impact of translation regulation of Mettl3 on beige fat biology.

## 4 Conclusion

In summary, we demonstrated a specific and critical role of Mettl3 in beige fat via its regulation in beige adipocyte glycolysis, which impacts beige fat thermogenesis and beige preadipocyte proliferation, therefore highlight the comprehensive role of Mettl3 in beige fat biology and systemic energy homeostasis.

## 5 Experimental Section

### Animals

Mice were allocated to experimental groups randomly. 8‐week male C57BL/6J mice (Jackson Laboratories) were used in the study. Mice with a targeted deletion of Mettl3 in adipose tissues (Adiponectin‐Cre Mettl3^loxp/loxp^) were generated by crossing the Mettl3^loxp/loxp^ mice with transgenic mice expressing Cre‐recombinase under the control of the adiponectin promoter (Adiponectin‐Cre). Littermates expressing no Cre (WT mice) were used as controls. Mice were housed in a temperature‐controlled room at 22 °C with a 12 h‐light/dark cycle and free access to food and water. For diet induced obesity studies, 8‐week‐old mice were fed HFD (ResearchDiet, D12492) for indicated time and monitored for changes in body weight, fat mass, cold tolerance, and oxygen consumption, then mice were sacrificed and tissues were dissected for further analysis. For cold stimulation and thermoneutral condition, mice were housed at 4 or 30 °C for 7 days in a temperature‐controlled chamber (NK system). Body weight, body composition, and food consumption were monitored every week throughout the experimental period. Mice were euthanized at the end of studies. Blood samples and tissue samples were then collected for analysis. All animal studies were carried out following the guidelines approved by the Ethics Committee of Animal Experiments of East China Normal University (m20211110).

### Surgical Removal of BAT

WT and Mettl3 FKO mice were received surgical removal of BAT. Under isoflurane‐inhalation anaesthetization, a small incision in the skin was performed along the upper dorsal surface in mice. The BAT pads were separated carefully from the surrounding tissues by blunt dissection using autoclaved surgical scissors, following with the closure of the incision with the sterile surgical suture. Blood vessels in the BAT were dissociated and ligated by absorbable surgical sutures to prevent excess bleeding after cutting out the tissues. All mice were allowed to recover for 1 week for the following experiments.

### MeRIP‐Seq, RNA‐Seq, and Data Analysis

For MeRIP‐Seq, methylated RNA immunoprecipitation sequencing was performed by Cloudseq Biotech Inc. (Shanghai, China) according to the published procedure with slight modifications. Briefly, 500 ng fragmented mRNAs were saved as input control for RNA‐Seq, 5 µg of fragmented mRNAs were incubated with 5 µg of anti‐m^6^A polyclonal antibody (Synaptic Systems, 202 003) in IP buffer (150 mm NaCl, 0.1% NP‐40, 10 mm Tris‐HCl, pH 7.4) for 2 h at 4 °C. The mixture was then immunoprecipitated by incubation with protein‐A beads (Thermo Fisher) at 4 °C for an additional 2 h. Then, bound mRNAs were eluted from the beads and then extracted with Trizol reagent (Thermo Fisher) by following the manufacturer's instruction. Purified mRNAs were used for RNA‐Seq library generation with NEBNext UltraTM RNA Library Prep Kit (NEB). Both the input sample (without immunoprecipitation) and the m^6^A IP sample were subjected to 150 bp paired‐end sequencing on Illumina HiSeq sequencer. Paired‐end reads were harvested from Illumina HiSeq 4000 sequencer, and qualification was controlled by Q30. After 3′adaptor‐trimming and low‐quality reads removing by cutadapt software (v1.9.3), the reads were aligned to the reference genome (UCSC MM10) with Hisat2 software (v2.0.4). RNA methylated sites (peaks) were identified by MACS2.^[^ ^55^ ^]^ HOMER (hypergeometric optimization of motif enRichment) was used to find motif sequence.^[^ ^56^ ^]^ Differentially methylated sites on RNAs were identified by diffReps.^[^ ^57^ ^]^

For RNA‐seq, RNA quality was examined with a Nanodrop spectrophotometer (Thermo, MA, USA) and agarose gel electrophoresis. High quality RNA was used to construct library and Illumina NovaSeq6000 was used to perform RNA‐seq (Genergy Biotechnology, Shanghai, China). Briefly, RNA libraries were constructed by TruSeq RNA LT Sample Prep Kit v2 (illumina, USA) following the manufacturer's protocol and quantified by Qubit (invitrogen, USA) for cluster generation. Processed RNA‐Seq data was filtered by removing genes with low read counts. Read counts were normalized using TMM normalization and CPM (counts per million) were calculated to create a matrix of normalized expression values. *p*‐value < 0.05 and |FC| > = 2 were used to determine differential genes. KEGG enrichment analysis was performed by clusterProfiler (v3.12.0). *p*‐value < 0.05 was considered as significantly enriched.

The *MeRIP‐Seq* and *RNA‐seq* datasets generated during this study are accessible at GEO: GSE222426.

### MeRIP‐qPCR

MeRIP assay was performed using MeRIP Kit (Epigenetics, P9018) following the manufacturer's instructions. Briefly, 10 µg of total RNAs were extracted from iWAT of cold and thermoneutral mice. Chemically fragmented RNA (about 200 nucleotides) was incubated with m^6^A antibody or non‐immune IgG‐conjugated beads in 200 µL IP buffer at room temperature for 90 min. Methylated RNA was immunoprecipitated, eluted, and recovered. Enrichment of m^6^A containing mRNA in each sample was analyzed by quantitative real‐time PCR and calculated by normalizing to input. The primer sequences were shown in Table S2, Supporting Information.

### m^6^A Quantification

The change of global m^6^A levels in mRNA was measured by m^6^A RNA methylation quantification kit (Colorimetric) (Epigenetics, P‐9005) following the manufacturer's protocol. Absorbance was measured at 450 nm using a microplate reader.

### Manipulation of Gene Expression Levels in Inguinal Fat or beige adipocytes

Adeno‐associated virus (AAV) vector‐mediated overexpression of mouse Mettl3, control (con), shRNA targeting Mettl3, scrambled control (shNC), Adenovirus (ADV) vector‐mediated overexpression of Mettl3, Igf2bp2, and control (Con) were constructed, amplified, and purified by Gene‐Chem (Shanghai, China). Besides, lentivirus vector‐mediated overexpression of Hk2, Pfkl, Pkm were constructed, amplified, and purified. For mice model, a total of 50 µL of 5 × 10^10^ Vg of each AAV diluted in PBS was injected into the inguinal fat pads of mice to manipulate the expression levels of specific genes. For in vitro experiments, preadipocytes or differentiated adipocytes were infected with ADV‐Mettl3, ADV‐Igf2bp2, lentivirus‐Hk2, Pfkl, Pkm, and their control to overexpression Mettl3, Igf2bp2, Hk2, Pfkl, and Pkm. To silence Mettl3 or Igf2bp2, siRNA targeting Mettl3 or Igf2bp2 and scramble control were transfected into preadipocytes or differentiated adipocytes using Lipofectamine2000 Transfection Reagent (Thermo Fisher, 11 668 030) according to the manufacturer's protocol. siRNA was designed and synthesized by GenePharma (Shanghai, China).

### Metabolic Measurements

Fat and lean mass were measured by body composition analyzer (Meg‐Med MRI system). Whole‐body energy expenditure (VO2, VCO2), food consumption and locomotor activity (beam break counts) were tracked using the comprehensive laboratory animal monitoring system (CLAMS).

For cold tolerance test, mice were housed at 4 °C and core temperature was measured at indicated time with a rectal thermometer (Braintree, TH‐5). For GTT experiments, mice were fasted overnight and then injected intraperitoneally (i.p.) with glucose solution in saline (1.5 g kg^−1^ body weight). For ITT experiments, the mice were injected i.p. with insulin (1.25 U kg^−1^ body weight, Sigma). Blood samples from tail vein were collected at indicated time points and glucose levels were measured with an AccuCheck blood glucose meter (Roche Diagnostics Inc.).

### SVF Isolation and Cell Sorting

SVFs were isolated as described previously.^[^ ^58^ ^]^ Briefly, inguinal fat from Mettl3 FKO or WT mice was minced and digested with 2 mg mL^−1^ collagenase type II (Sigma, C6885) in PBS supplemented with 1% Hepes at 37 °C for 20–30 min, followed by quenching with complete medium. Cell suspensions were centrifuged, washed, and filtered through a 40‐µm strainer. For fluorescence activated cell sorting (FACS) analysis, SVFs were resuspended in red blood cell lysis buffer (Beyotime, C3702) for 15 min at room temperature and then further incubated with PE anti‐mouse Pdgfr*α* (BioLegend, 135 905), APC anti‐mouse CD31 (BioLegend, 102 509), APC anti‐mouse CD45 (BioLegend, 103 116), and subjected to FACS.

### Cell Culture and Beige Adipocyte Differentiation

Immortalized beige adipocytes and primary iWAT SVFs were plated in Dulbecco's Modified Eagle's Medium (DMEM, Gibco, 11 995 065), supplemented with 20% FBS (Gibco, 10 270), and 1% streptomycin and penicillin (Gibco, 15 070 063). For beige adipocyte differentiation, immortalized beige preadipocytes were induced in differentiation medium supplemented with 5 µg mL^−1^ insulin (Sigma, I9278), 0.5 mm IBMX (Sigma, I5879), 1 µm dexamethasone (Sigma, D4902), and 1 µm rosiglitazone (Sigma, R2408) for 2 days and subsequently cultured in maintenance medium supplemented with 1 µm rosiglitazone and 5 µg mL^−1^ insulin. Primary iWAT SVFs were induced to differentiate into beige adipocytes with an adipogenic cocktail (6 µg mL^−1^ insulin, 0.5 mm IBMX, 1 µm dexamethasone, 50 nm T3, and 1 µm rosiglitazone) in DMEM medium containing 10% FBS, 1% pen/strep for 2 days and subsequently maintained in maintenance medium containing 50 nm T3, 1 µm rosiglitazone and 6 µg mL^−1^ insulin. The maintenance medium was changed every two days and mature adipocytes were collected on seventh day. Cells were cultured at 37 °C in a humidified incubator of 5% CO_2_. To investigate cellular thermogenesis, differentiated adipocytes were treated with ERthermAC dye (250 nm, Emdmillipore, SCT057) for 30 min and subjected to FACS analysis (BD LSR Fortessainstrument).

### RNA Isolation and Quantitative RT‐PCR

Total RNA was extracted from isolated adipocytes or adipose tissue samples using TRIZOL Reagent (TaKaRa, 9108). For RT‐PCR, 1 µg of total RNA was reverse‐transcribed to cDNA using the PrimeScriptTM RT Master Mix (TaKaRa, RR036A). Quantitative PCR was performed in a LightCycler 480 II (Roche) using HieffTM qPCR SYBR Green Master Mix (Low Rox Plus) (Yeasen, 11202ES03). 36B4 was used as reference gene in data analysis. The primer sequences used are listed in Table S2, Supporting Information.

### RNA Immunoprecipitation (RIP) Assay

RIP assay was performed using Magna RIP RNA‐binding protein immunoprecipitation kit according to the manufacturer's instructions (RN1001, MBL). Briefly, fully differentiated beige adipocytes were washed with cold PBS followed by lysed in RIP lysis buffer containing RNase inhibitor. The lysates were incubated with anti‐IGF2BP2 antibody (Proteintech, 11601‐1‐AP) and Sepharose beads overnight at 4 °C. After extensive washing with RIP washing buffer (50 mm Tris‐HCl pH 7.5, 500 mm NaCl, 4 mm MgCl2, 5 mm DTT, 0.5% Igepal CA‐630, 1% SDS, 0.5% sodium deoxycholate, 2 m urea), co‐precipitated RNAs were eluted with TriReagent and treated with DNase (Promega). The RNA samples precipitated were extracted, reverse transcribed, and subjected to RT‐qPCR analysis.

### Oxygen Consumption Rate and Extracellular Acidification Rate Measurements

Oxygen consumption rate (OCR) and extracellular acidification rate (ECAR) of beige adipocytes were measured using the XF24 analyzer (Seahorse Bioscience) as previously described.^[^ ^59^ ^]^ All compounds and media were prepared according to the manufacturer's instructions. In brief, OCR was measured in XF assay medium supplemented with sodium pyruvate (2 mm), l‐glutamine (LG, 2 mm), and glucose (25 mm) under basal conditions and in response to oligomycin (1 µm), carbonyl cyanide‐*p*‐trifluoromethoxyphenylhydrazone (FCCP, 2 µm), antimycin A (0.5 µm), and rotenone (0.5 µm). ECAR was measured in XF assay medium supplemented with LG (2 mm) in basal conditions and in response to glucose (10 mm) and 2‐deoxyglucose (2‐DG, 0.1 m). The OCR and ECAR values were normalized to protein content.

### Glucose Uptake Assay

iWAT glucose uptake was measured by the uptake of 2‐deoxy‐d‐[^14^C] glucose (PerkinElmer, NEC720A250UC). Mice were injected intraperitoneally with 2‐deoxy‐d‐[^14^C] glucose (50 µCi kg^−1^) and sacrificed 30 min later. iWATs were harvested, weighed, and immediately digested in NaOH (1 m, 1 mL per 100 mg tissue) at 60 °C for 2 h followed by addition of HCL (2 m, 0.5 mL per 1 mL NaOH) and centrifuged. The radioactivity of supernatant was measured by a Liquid Scintillation Counter (Tri‐Carb 4910TR). The glucose uptake was normalized by protein concentrations as quantified by BCA method.

### Measurements of Lactate and Pyruvate Production

Adipose tissues were homogenized in ice‐cold PBS followed by centrifugation at 2500 rpm for 10 min. Lactate and pyruvate levels in supernatants were determined by lactic acid assay kit and pyruvate assay kit following the manufacturer's protocols (Nanjing Jiancheng).

### Histological Analysis

Adipose and liver tissues were fixed in 10% formalin for 12–24 h, embedded in paraffin, and sectioned. Sections were stained with Hematoxylin and eosin (Beyotime, C0105S) according to the manufacturer's instructions. The images were acquired by optical microscope (Nikon) using a 20× objective. Adipocyte sizes were quantified by ImageJ and AdipoCount. For UCP1 IHC staining, 5µm‐ thick iWAT sections were pre‐incubated with permeabilization buffer (Beyotime) for 30 min at room temperature and then incubated with UCP1 antibody (Abcam, ab10983) in blocking buffer (5% normal goat serum in PBS) at 4 °C overnight. Antigens were detected using primary antibody and in conjunction with a DAB chromogenic detection kit (ZSGB‐BIO, ZLI‐9018) according to the manufacturer's instructions. Immunostained images were collected on optical microscope (Nikon).

### Immunoblotting

Cells and tissues were lysed in RIPA lysis buffer (Thermo Fisher, 89 900) containing PMSF (Thermo Fisher, 36 978) and protease inhibitors. Total protein lysates were boiled with loading sample buffer containing 10% SDS‐PAGE. Subsequently, separated proteins were transferred onto PVDF membranes. PVDF membrane blots were blocked in 10% skimmed milk for 1 h at room temperature, washed in Tris‐buffered saline with Tween 20 (TBS‐T), and incubated overnight at 4 °C with rabbit anti‐METTL3 (Proteintech, 15073‐1‐AP), rabbit anti‐PDGFR*α* (CST, 3174), and mouse anti‐*β*‐ACTIN (Sigma‐Aldrich, A3854). Anti‐rabbit IgG (LI‐COR, 926–68071) was used as the second antibody for METTL3 and PDGFR*α*. Anti‐mouse IgG (LI‐COR, 926–32210) was used as the secondary antibody for *β*‐ACTIN.

### Immunofluorescence

Immunostaining was performed in paraffin sections according to the standard protocols. In brief, samples were deparaffinized and rehydrated, followed by antigen retrieval in 10 mm sodium citrate (Beyotime, P0083). Blocking and staining were performed in antibody diluent with 10% Goat serum. Sections were incubated with mouse anti‐Pdgfr*α* (Santa Cruz, sc‐398206) and rabbit anti‐Ki67 (Bethyl Laboratories, IHC‐00375) antibodies overnight at 4 °C, followed by incubation with the corresponding secondary antibodies goat anti‐mouse Alexa Fluor 594 (Thermo Fisher, A11032) and anti‐rabbit Alexa Fluor 488 (Thermo Fisher, A11034), respectively. Slides were mounted with DAPI (4′,6‐diamidino‐2‐phenylindole) and imaged by fluorescence microscopy. Cell counting was achieved using the ImageJ software.

### RNA Stability Assay

Differentiated immortalized beige adipocytes were transfected with siMettl3/siIgf2bp2/Scramble (scr) or ADV‐Mettl3/ADV‐Igf2bp2/ADV‐Control (Con), treated with 5 µg mL^−1^ actinomycin D (Sigma, SBR00013) for transcription inhibition and harvested at indicated time points. The total RNA was isolated and analyzed by qRT‐PCR.

The degradation rate of mRNA *K*_d_ was estimated by: ln (*C*_t_/*C*_0_) = −*K*_d_ *t*, where *t* is the time after actinomycin D treatment. *C* is the mRNA concentration. The mRNA lifetime *t*_1/2_ is calculated by: *t*_1/2_ = ln2/*K*_d_

### Plasmid Construction and Luciferase Reporter Assays

The Igf2bp2‐CDS was amplified from mouse genomic cDNA, and directly inserted into pCDH vector. The DNA fragments of Hk2, Pfkl, Pkm 3′UTR containing the wild‐type m^6^A motifs and mutant motifs (A was replaced by T) were inserted into downstream of firefly luciferase of pMIR‐REPORT vector (Ambion). For dual‐luciferase reporter assays, HEK293T cells were co‐transfected with wild‐type/mutant Hk2, Pfkl, and Pkm 3′UTR reporter plasmids, or Igf2bp2 expression plasmid/control plasmid with EZ‐Trans transfection reagent (Life Ilab Biotechnology, C4058L1090). Cells were harvested with passive lysis buffer (Promega, E1960) 24 h after transfection and luciferase activity was measured using the Dual‐Luciferase Reporter Assay System (Promega, E1960).

### Conditioned Medium Preparation and Cell Proliferation Assay

For conditioned medium preparation, differentiated immortalized beige adipocytes or primary adipocytes were transfected with siMettl3/siIgf2bp2/scramble or ADV‐Mettl3/ADV‐Igf2bp2/ADV‐Con or lentivirus‐Hk2/Pfkl/Pkm followed by switching to fresh DMEM medium containing 10% FBS for 24 h. Then the conditioned medium from mature adipocytes was collected. For CCK8 assay, the preadipocytes were seeded into a 96‐well plate. After 24 h, cells were treated with conditioned medium from mature adipocytes for 24 h. CCK8 reagent (Beyotime, C0037) was added to each well and optical density value was detected using a microplate reader at 450 nm for further statistical analysis. For EdU assay, preadipocytes were cultured in 48‐well plates, incubated with 10 µm EdU for 2 h, and then washed, fixed, neutralized, and permeabilized according to manufacturer's protocol (Beyotime, C0071S). The nuclei were stained with Hoechst for 10 min. The stained preadipocytes were observed using microscope (Nikon) and the data were analyzed using ImageJ.

### Statistical Analysis

Statistics analysis were performed using GraphPad Prism 7 software. The statistical details of experiment are indicated in the Figure legend. Statistical comparisons between two groups were made by unpaired Student's *t*‐test. Two‐way ANOVA with Bonferroni's multiple comparisons was used for comparisons of multiple factors and ANCOVA was used to analyze oxygen consumption data by SPSS software. Results are expressed as mean ± SEM. *p* < 0.05 was considered as statistically significant, *p < 0.05, **p < 0.01.

## Conflict of Interest

The authors declare no conflict of interest.

## Author Contributions

Y.L., Y.Z., and T.Z. contributed equally to this work. X.M. conceptualized and supervised the study. Y.L., Y.Z., and T.Z. developed the experimental methods. X.P., D.W., Y.C., J.Y., C.L., Z.L., Y.Z., Y.Y., C.R., D.L., Z.D., and J.W. performed the investigations and data analyses. Y.L., Y.Z., and T.Z. wrote the original draft of the manuscript. X.M. and L.X. reviewed and edited the manuscript.

## Supporting information

Supporting Information

Supplemental Table 1

## Data Availability

The data that support the findings of this study are available from the corresponding author upon reasonable request.

## References

[1] C. Ji , X. Guo , Nat. Rev. Endocrinol. 2019, 15, 731. 31611648 10.1038/s41574-019-0260-0

[2] T. T. Tran , C. R. Kahn , Nat. Rev. Endocrinol. 2010, 6, 195. 20195269 10.1038/nrendo.2010.20 PMC4362513

[3] M. T. Hyvonen , K. L. Spalding , Int. J. Biochem. Cell Biol. 2014, 56, 123. 25240584 10.1016/j.biocel.2014.09.013

[4] M. Harms , P. Seale , Nat. Med. 2013, 19, 1252. 24100998 10.1038/nm.3361

[5] Y. Li , D. Wang , X. Ping , Y. Zhang , T. Zhang , L. Wang , L. Jin , W. Zhao , M. Guo , F. Shen , M. Meng , X. Chen , Y. Zheng , J. Wang , D. Li , Q. Zhang , C. Hu , L. Xu , X. Ma , Cell 2022, 185, 949. 35247329 10.1016/j.cell.2022.02.004

[6] W. D. van Marken Lichtenbelt , J. W. Vanhommerig , N. M. Smulders , J. M. Drossaerts , G. J. Kemerink , N. D. Bouvy , P. Schrauwen , G. J. Teule , N. Engl. J. Med. 2009, 360, 1500. 19357405 10.1056/NEJMoa0808718

[7] K. A. Virtanen , M. E. Lidell , J. Orava , M. Heglind , R. Westergren , T. Niemi , M. Taittonen , J. Laine , N. J. Savisto , S. Enerback , P. Nuutila , N. Engl. J. Med. 2009, 360, 1518. 19357407 10.1056/NEJMoa0808949

[8] Y. Chen , K. Ikeda , T. Yoneshiro , A. Scaramozza , K. Tajima , Q. Wang , K. Kim , K. Shinoda , C. H. Sponton , Z. Brown , A. Brack , S. Kajimura , Nature 2019, 565, 180. 30568302 10.1038/s41586-018-0801-z PMC6328316

[9] S. Yan , M. Kumari , H. Xiao , C. Jacobs , S. Kochumon , M. Jedrychowski , E. Chouchani , R. Ahmad , E. D. Rosen , J. Clin. Invest. 2021, 131, e144888. 33571167 10.1172/JCI144888 PMC8011904

[10] X. W. Jia , D. L. Fang , X. Y. Shi , T. Lu , C. Yang , Y. Gao , Biochim. Biophys. Acta, Mol. Cell Biol. Lipids 2021, 1866, 158871. 33346159 10.1016/j.bbalip.2020.158871

[11] P. Cohen , J. D. Levy , Y. Zhang , A. Frontini , D. P. Kolodin , K. J. Svensson , J. C. Lo , X. Zeng , L. Ye , M. J. Khandekar , J. Wu , S. C. Gunawardana , A. S. Banks , J. P. Camporez , M. J. Jurczak , S. Kajimura , D. W. Piston , D. Mathis , S. Cinti , G. I. Shulman , P. Seale , B. M. Spiegelman , Cell 2014, 156, 304. 24439384 10.1016/j.cell.2013.12.021 PMC3922400

[12] S. Kleiner , R. J. Mepani , D. Laznik , L. Ye , M. J. Jurczak , F. R. Jornayvaz , J. L. Estall , D. Chatterjee Bhowmick , G. I. Shulman , B. M. Spiegelman , Proc. Natl. Acad. Sci. U. S. A. 2012, 109, 9635. 22645355 10.1073/pnas.1207287109 PMC3386123

[13] X. Zeng , M. P. Jedrychowski , Y. Chen , S. Serag , G. G. Lavery , S. P. Gygi , B. M. Spiegelman , Genes Dev. 2016, 30, 1822. 27566776 10.1101/gad.285312.116 PMC5024681

[14] H. Ohno , K. Shinoda , K. Ohyama , L. Z. Sharp , S. Kajimura , Nature 2013, 504, 163. 24196706 10.1038/nature12652 PMC3855638

[15] C. Zhang , Y. Chen , B. Sun , L. Wang , Y. Yang , D. Ma , J. Lv , J. Heng , Y. Ding , Y. Xue , X. Lu , W. Xiao , Y. G. Yang , F. Liu , Nature 2017, 549, 273. 28869969 10.1038/nature23883

[16] H. Shi , X. Wang , Z. Lu , B. S. Zhao , H. Ma , P. J. Hsu , C. Liu , C. He , Cell Res. 2017, 27, 315. 28106072 10.1038/cr.2017.15 PMC5339834

[17] J. Kretschmer , H. Rao , P. Hackert , K. E. Sloan , C. Hobartner , M. T. Bohnsack , RNA 2018, 24, 1339. 29970596 10.1261/rna.064238.117 PMC6140455

[18] Y. Fu , D. Dominissini , G. Rechavi , C. He , Nat. Rev. Genet. 2014, 15, 293. 24662220 10.1038/nrg3724

[19] X. Jiang , B. Liu , Z. Nie , L. Duan , Q. Xiong , Z. Jin , C. Yang , Y. Chen , Signal Transduction Targeted Ther. 2021, 6, 74. 10.1038/s41392-020-00450-x PMC7897327 33611339

[20] D. F. De Jesus , Z. Zhang , S. Kahraman , N. K. Brown , M. Chen , J. Hu , M. K. Gupta , C. He , R. N. Kulkarni , Nat. Metab. 2019, 1, 765. 31867565 10.1038/s42255-019-0089-9 PMC6924515

[21] B. Zhou , C. Liu , L. Xu , Y. Yuan , J. Zhao , W. Zhao , Y. Chen , J. Qiu , M. Meng , Y. Zheng , D. Wang , X. Gao , X. Li , Q. Zhao , X. Wei , D. Wu , H. Zhang , C. Hu , X. Zhuo , M. Zheng , H. Wang , Y. Lu , X. Ma , Hepatology 2021, 73, 91. 32150756 10.1002/hep.31220

[22] Y. Qin , B. Li , S. Arumugam , Q. Lu , S. M. Mankash , J. Li , B. Sun , J. Li , R. A. Flavell , H. B. Li , X. Ouyang , Cell Rep. 2021, 37, 109968. 34758326 10.1016/j.celrep.2021.109968 PMC8667589

[23] S. K. Azzam , H. Alsafar , A. A. Sajini , Int. J. Mol. Sci. 2022, 23, 3800. 35409166 10.3390/ijms23073800 PMC8998816

[24] Y. Wang , M. Gao , F. Zhu , X. Li , Y. Yang , Q. Yan , L. Jia , L. Xie , Z. Chen , Nat. Commun. 2020, 11, 1648. 32245957 10.1038/s41467-020-15488-2 PMC7125133

[25] J. Zhong , Q. Kang , Y. Cao , B. He , P. Zhao , Y. Gou , Y. Luo , T. C. He , J. Fan , Am. J. Cancer Res. 2021, 11, 793. 33791154 PMC7994163

[26] T. Seki , Y. Yang , X. Sun , S. Lim , S. Xie , Z. Guo , W. Xiong , M. Kuroda , H. Sakaue , K. Hosaka , X. Jing , M. Yoshihara , L. Qu , X. Li , Y. Chen , Y. Cao , Nature 2022, 608, 421. 35922508 10.1038/s41586-022-05030-3 PMC9365697

[27] H. P. Nguyen , D. Yi , F. Lin , J. A. Viscarra , C. Tabuchi , K. Ngo , G. Shin , A. Y. Lee , Y. Wang , H. S. Sul , Mol. Cell 2020, 77, 600. 31952989 10.1016/j.molcel.2019.12.002 PMC7031813

[28] Y. Xu , T. Shi , X. Cui , L. Yan , Q. Wang , X. Xu , Q. Zhao , X. Xu , Q. Q. Tang , H. Tang , D. Pan , EMBO J. 2021, 40, 108069. 10.15252/embj.2021108069 PMC8672174 34704268

[29] H. J. Wang , C. S. Lee , R. S. Z. Yee , L. Groom , I. Friedman , L. Babcock , D. K. Georgiou , J. Hong , A. D. Hanna , J. Recio , J. M. Choi , T. Chang , N. H. Agha , J. Romero , P. Sarkar , N. Voermans , M. W. Gaber , S. Y. Jung , M. L. Baker , R. G. Pautler , R. T. Dirksen , S. Riazi , S. L. Hamilton , Nat. Commun. 2020, 11, 5099. 33037202 10.1038/s41467-020-18865-z PMC7547078

[30] J. N. Wang , F. Wang , J. Ke , Z. Li , C. H. Xu , Q. Yang , X. Chen , X. Y. He , Y. He , X. G. Suo , C. Li , J. T. Yu , L. Jiang , W. J. Ni , J. Jin , M. M. Liu , W. Shao , C. Yang , Q. Gong , H. Y. Chen , J. Li , Y. G. Wu , X. M. Meng , Sci. Transl. Med. 2022, 14, eabk2709. 35417191 10.1126/scitranslmed.abk2709

[31] C. Shen , B. Xuan , T. Yan , Y. Ma , P. Xu , X. Tian , X. Zhang , Y. Cao , D. Ma , X. Zhu , Y. Zhang , J. Y. Fang , H. Chen , J. Hong , Mol. Cancer 2020, 19, 72. 32245489 10.1186/s12943-020-01190-w PMC7118901

[32] H. Liu , S. Qin , C. Liu , L. Jiang , C. Li , J. Yang , S. Zhang , Z. Yan , X. Liu , J. Yang , X. Sun , Cell Death Discovery 2021, 7, 292. 34645788 10.1038/s41420-021-00674-y PMC8514511

[33] Y. Zhou , P. Zeng , Y. H. Li , Z. Zhang , Q. Cui , Nucleic Acids Res. 2016, 44, e91. 26896799 10.1093/nar/gkw104 PMC4889921

[34] X. Kong , A. Banks , T. Liu , L. Kazak , R. R. Rao , P. Cohen , X. Wang , S. Yu , J. C. Lo , Y. H. Tseng , A. M. Cypess , R. Xue , S. Kleiner , S. Kang , B. M. Spiegelman , E. D. Rosen , Cell 2014, 158, 69. 24995979 10.1016/j.cell.2014.04.049 PMC4116691

[35] Y. Chen , Z. Wu , S. Huang , X. Wang , S. He , L. Liu , Y. Hu , L. Chen , P. Chen , S. Liu , S. He , B. Shan , L. Zheng , S. Z. Duan , Z. Song , L. Jiang , Q. A. Wang , Z. Gan , B. L. Song , J. Liu , L. Rui , M. Shao , Y. Liu , Nat. Metab. 2022, 4, 1166. 36123394 10.1038/s42255-022-00631-8

[36] K. Y. Lee , S. J. Russell , S. Ussar , J. Boucher , C. Vernochet , M. A. Mori , G. Smyth , M. Rourk , C. Cederquist , E. D. Rosen , B. B. Kahn , C. R. Kahn , Diabetes 2013, 62, 864. 23321074 10.2337/db12-1089 PMC3581196

[37] S. Kang , X. Kong , E. D. Rosen , Methods Enzymol. 2014, 537, 1. 24480338 10.1016/B978-0-12-411619-1.00001-X

[38] R. Xie , S. Yan , X. Zhou , Y. Gao , Y. Qian , J. Hou , Z. Chen , K. Lai , X. Gao , S. Wei , Diabetes 2023, db220775. 10.2337/db22-0775 37224383

[39] J. Wu , P. Bostrom , L. M. Sparks , L. Ye , J. H. Choi , A. H. Giang , M. Khandekar , K. A. Virtanen , P. Nuutila , G. Schaart , K. Huang , H. Tu , W. D. van Marken Lichtenbelt , J. Hoeks , S. Enerback , P. Schrauwen , B. M. Spiegelman , Cell 2012, 150, 366. 22796012 10.1016/j.cell.2012.05.016 PMC3402601

[40] C. Y. Wang , S. S. Shie , M. S. Wen , K. C. Hung , I. C. Hsieh , T. S. Yeh , D. Wu , Sci. Signaling 2015, 8, ra127. 10.1126/scisignal.aab3357 26671148

[41] E. D. Rosen , B. M. Spiegelman , Cell 2014, 156, 20.24439368

[42] L. Scheja , J. Heeren , Nat Rev Endocrinol 2019, 15, 507. 31296970 10.1038/s41574-019-0230-6

[43] X. Wang , R. Wu , Y. Liu , Y. Zhao , Z. Bi , Y. Yao , Q. Liu , H. Shi , F. Wang , Y. Wang , Autophagy 2020, 16, 1221. 31451060 10.1080/15548627.2019.1659617 PMC7469583

[44] M. Kobayashi , M. Ohsugi , T. Sasako , M. Awazawa , T. Umehara , A. Iwane , N. Kobayashi , Y. Okazaki , N. Kubota , R. Suzuki , H. Waki , K. Horiuchi , T. Hamakubo , T. Kodama , S. Aoe , K. Tobe , T. Kadowaki , K. Ueki , Mol. Cell. Biol. 2018, 38, e00116. 29866655 10.1128/MCB.00116-18 PMC6066751

[45] D. Tews , P. Fischer‐Posovszky , T. Fromme , M. Klingenspor , J. Fischer , U. Ruther , R. Marienfeld , T. F. Barth , P. Moller , K. M. Debatin , M. Wabitsch , Endocrinology 2013, 154, 3141. 23751871 10.1210/en.2012-1873

[46] M. G. Vander Heiden , L. C. Cantley , C. B. Thompson , Science 2009, 324, 1029. 19460998 10.1126/science.1160809 PMC2849637

[47] Z. Li , Y. Peng , J. Li , Z. Chen , F. Chen , J. Tu , S. Lin , H. Wang , Nat. Commun. 2020, 11, 2578. 32444598 10.1038/s41467-020-16306-5 PMC7244544

[48] H. Shin , Y. Ma , T. Chanturiya , Q. Cao , Y. Wang , A. K. G. Kadegowda , R. Jackson , D. Rumore , B. Xue , H. Shi , O. Gavrilova , L. Yu , Cell Metab. 2017, 26, 764. 28988822 10.1016/j.cmet.2017.09.002 PMC5905336

[49] S. Winther , M. S. Isidor , A. L. Basse , N. Skjoldborg , A. Cheung , B. Quistorff , J. B. Hansen , Am. J. Physiol. Endocrinol. Metab. 2018, 314, E214. 29118013 10.1152/ajpendo.00218.2017

[50] D. C. Berry , Y. Jiang , J. M. Graff , Trends Endocrinol. Metab. 2016, 27, 574. 27262681 10.1016/j.tem.2016.05.001 PMC10947416

[51] N. Dai , L. Zhao , D. Wrighting , D. Kramer , A. Majithia , Y. Wang , V. Cracan , D. Borges‐Rivera , V. K. Mootha , M. Nahrendorf , D. R. Thorburn , L. Minichiello , D. Altshuler , J. Avruch , Cell Metab. 2015, 21, 609. 25863250 10.1016/j.cmet.2015.03.006 PMC4663978

[52] J. R. Alvarez‐Dominguez , S. Winther , J. B. Hansen , H. F. Lodish , M. Knoll , iScience 2022, 25, 103680. 35036870 10.1016/j.isci.2021.103680 PMC8749451

[53] X. Lin , G. Chai , Y. Wu , J. Li , F. Chen , J. Liu , G. Luo , J. Tauler , J. Du , S. Lin , C. He , H. Wang , Nat. Commun. 2019, 10, 2065. 31061416 10.1038/s41467-019-09865-9 PMC6502834

[54] L. Liu , Y. Wu , Q. Li , J. Liang , Q. He , L. Zhao , J. Chen , M. Cheng , Z. Huang , H. Ren , J. Chen , L. Peng , F. Gao , D. Chen , A. Wang , Mol. Ther. 2020, 28, 2177. 32621798 10.1016/j.ymthe.2020.06.024 PMC7544972

[55] Y. Zhang , T. Liu , C. A. Meyer , J. Eeckhoute , D. S. Johnson , B. E. Bernstein , C. Nusbaum , R. M. Myers , M. Brown , W. Li , X. S. Liu , Genome Biol. 2008, 9, R137. 18798982 10.1186/gb-2008-9-9-r137 PMC2592715

[56] S. H. Duttke , M. W. Chang , S. Heinz , C. Benner , Genome Res. 2019, 29, 1836. 31649059 10.1101/gr.253492.119 PMC6836739

[57] L. Shen , N. Y. Shao , X. Liu , I. Maze , J. Feng , E. J. Nestler , PLoS One 2013, 8, e65598. 23762400 10.1371/journal.pone.0065598 PMC3677880

[58] A. Vegiopoulos , K. Muller‐Decker , D. Strzoda , I. Schmitt , E. Chichelnitskiy , A. Ostertag , M. Berriel Diaz , J. Rozman , M. Hrabe de Angelis , R. M. Nusing , C. W. Meyer , W. Wahli , M. Klingenspor , S. Herzig , Science 2010, 328, 1158. 20448152 10.1126/science.1186034

[59] V. Sukonina , H. Ma , W. Zhang , S. Bartesaghi , S. Subhash , M. Heglind , H. Foyn , M. J. Betz , D. Nilsson , M. E. Lidell , J. Naumann , S. Haufs‐Brusberg , H. Palmgren , T. Mondal , M. Beg , M. P. Jedrychowski , K. Tasken , A. Pfeifer , X. R. Peng , C. Kanduri , S. Enerback , Nature 2019, 566, 279. 30700909 10.1038/s41586-019-0900-5

