# Supplementary material for: Rna M6a Methylation Regulates Glycolysis of Beige Fat and Contributes to Systemic Metabolic Homeostasis
Source: Adv Sci (Weinh). 2023 Jul 5;10(25):2300436. doi: 10.1002/advs.202300436 (PMC10477848; doi:10.1002/advs.202300436)
Supplement: Supplementary file 1 — Supporting Information [file ADVS-10-2300436-s001.pdf]

ADVANCED SCIENCE
Open Access

# Supporting Information

for *Adv. Sci.*, DOI 10.1002/advs.202300436

Rna $M^6a$ Methylation Regulates Glycolysis of Beige Fat and Contributes to Systemic Metabolic Homeostasis

*Yu Li, Yankang Zhang, Ting Zhang, Xiaodan Ping, Dongmei Wang, Yanru Chen, Jian Yu, Caizhi Liu, Ziqi Liu, Yuhan Zheng, Yongfeng Yang, Chengchao Ruan, Dali Li, Zhenyu Du, Jiqiu Wang, Lingyan Xu and Xinran Ma**

Supporting Information Figure Captions:

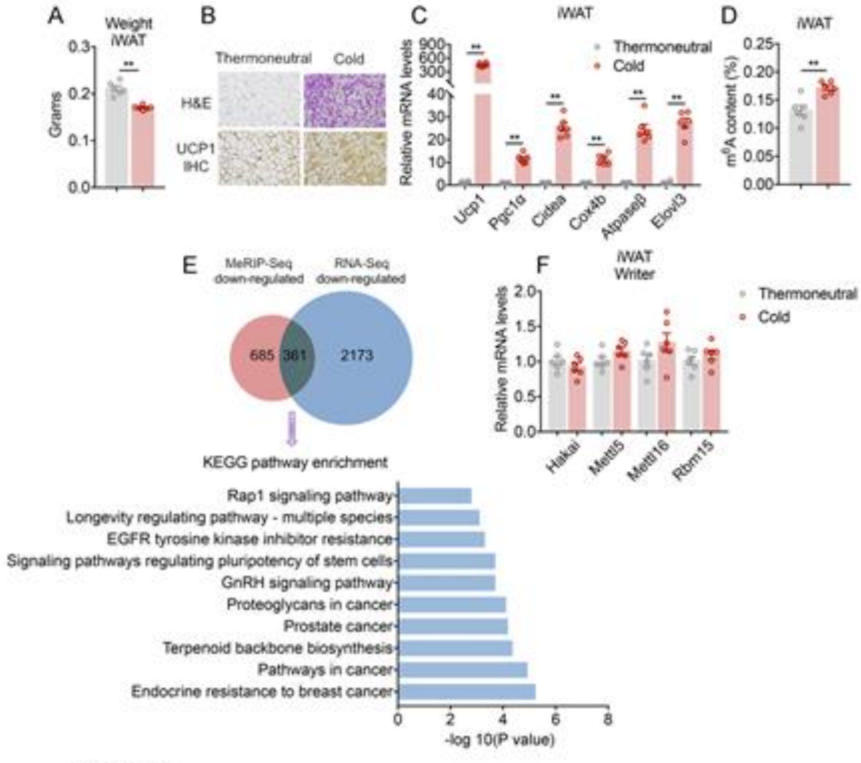


Figure S1

Supplementary Figure 1. The phenotype of mice at thermoneutral or cold exposed for 7 days.

A. Weight of inguinal white adipose tissues (iWAT) from mice under thermoneutral or cold (n=6).

B. H&E staining and UCP1 IHC of iWAT from mice under thermoneutral or cold.

C. Relative mRNA levels of brown gene programs in iWAT from mice under thermoneutral or cold (n=6).

D. The m6A contents of total RNAs in the iWAT from mice under chronic cold stimulation or thermoneutral condition (n=6).

E. Venn diagram and KEGG analysis overlapped genes with decreased m6A modification in MeRIP-seq and down-regulated mRNA levels in RNA-seq in iWAT from mice under chronic cold stimulation or thermoneutral condition.

F. Relative mRNA levels of various m6A writer including Hakai, Mettl5, Mettl16 and Rbm15 in iWAT from mice under chronic cold stimulation or thermoneutral condition (n=6).

Data are shown as mean ± SEM. Statistical significance was analyzed by unpaired Student's t-test (A, C, D, F). *$P < 0.05$, **$P < 0.01$. Scale bars, 50μm.

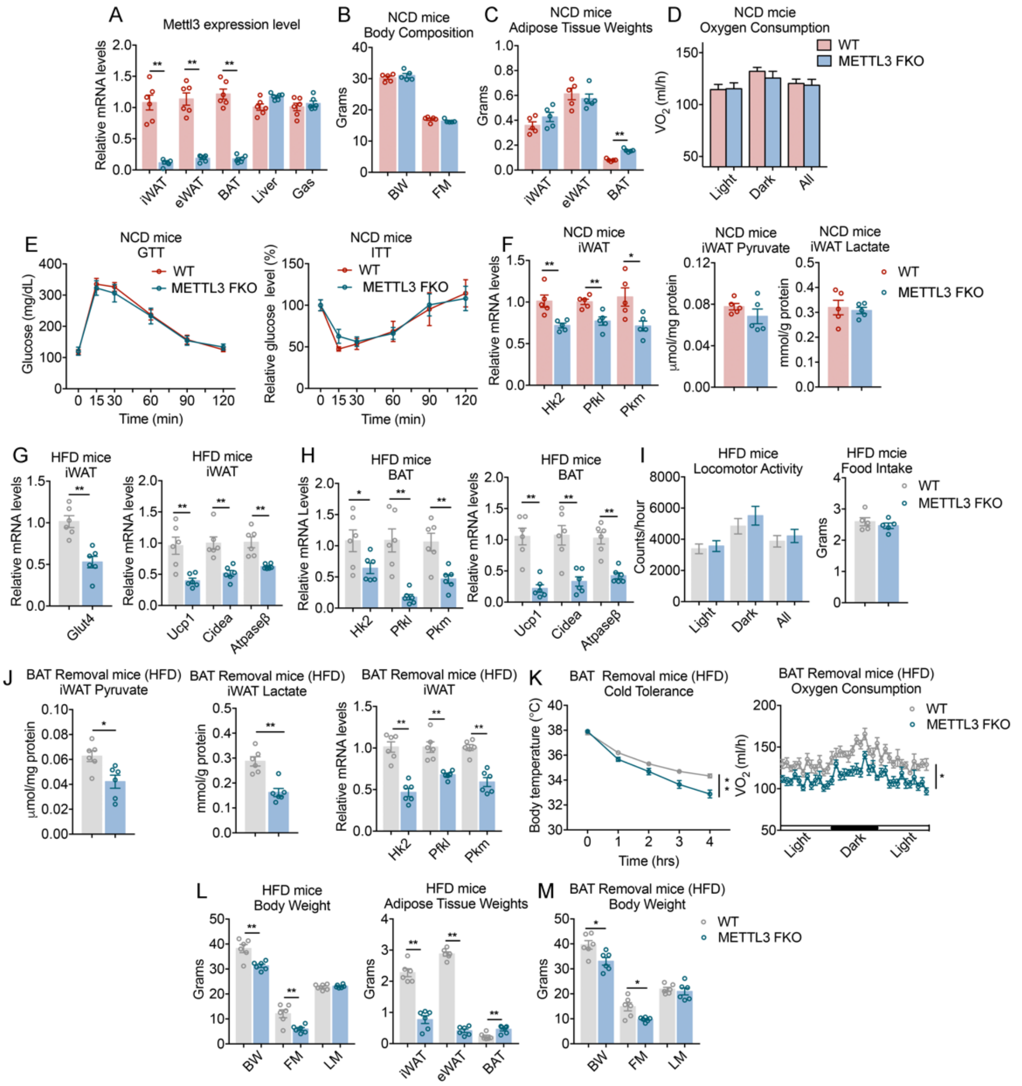


Figure S2

Supplementary Figure 2. Metabolic phenotyping of WT and Mettl3 FKO mice on NCD and HFD.

A. Mettl3 expression in metabolic organs in WT and Mettl3 FKO mice (n=6).

B-E. Analysis of metabolic parameters of WT and Mettl3 FKO mice on

NCD, including (B) body weight (BW), fat mass (FM); (C) adipose tissue weights; (D) oxygen consumption; (E) GTT and ITT (n=5).

F. The relative mRNA levels of glycolytic genes and the levels of pyruvate and lactate in iWAT of WT and Mettl3 FKO on NCD (n=5).

G-I, L. Analysis of metabolic parameters of WT and Mettl3 FKO mice on HFD, including (G) Relative mRNA levels of Glut4 and thermogenic genes in iWAT of WT and Mettl3 FKO on HFD (n=6); (H) Relative mRNA levels of glycolytic and thermogenic genes in BAT; (I) locomotor activity and food intake (n=5); (L) body weight, body composition and adipose tissue weights (n=6).

J, K, M. Analysis of metabolic parameters of WT and Mettl3 FKO mice after removal of BAT on HFD, including: (J) The levels of pyruvate and lactate; relative mRNA levels of glycolytic genes in iWAT (n=6); (K) oxygen consumption (n=5) and cold tolerance (n=6); (M) Body weight (BW) , fat mass (FM) and lean mass (LM) (n=6).

Data are shown as mean ± SEM. Statistical significance was analyzed by unpaired Student’s t-test (A-C, F-J, L-M), two-way ANOVA followed with Bonferroni’s multiple comparisons test (E, K) or ANCOVA with body weight as covariant (D, K). *$P < 0.05$, **$P < 0.01$.

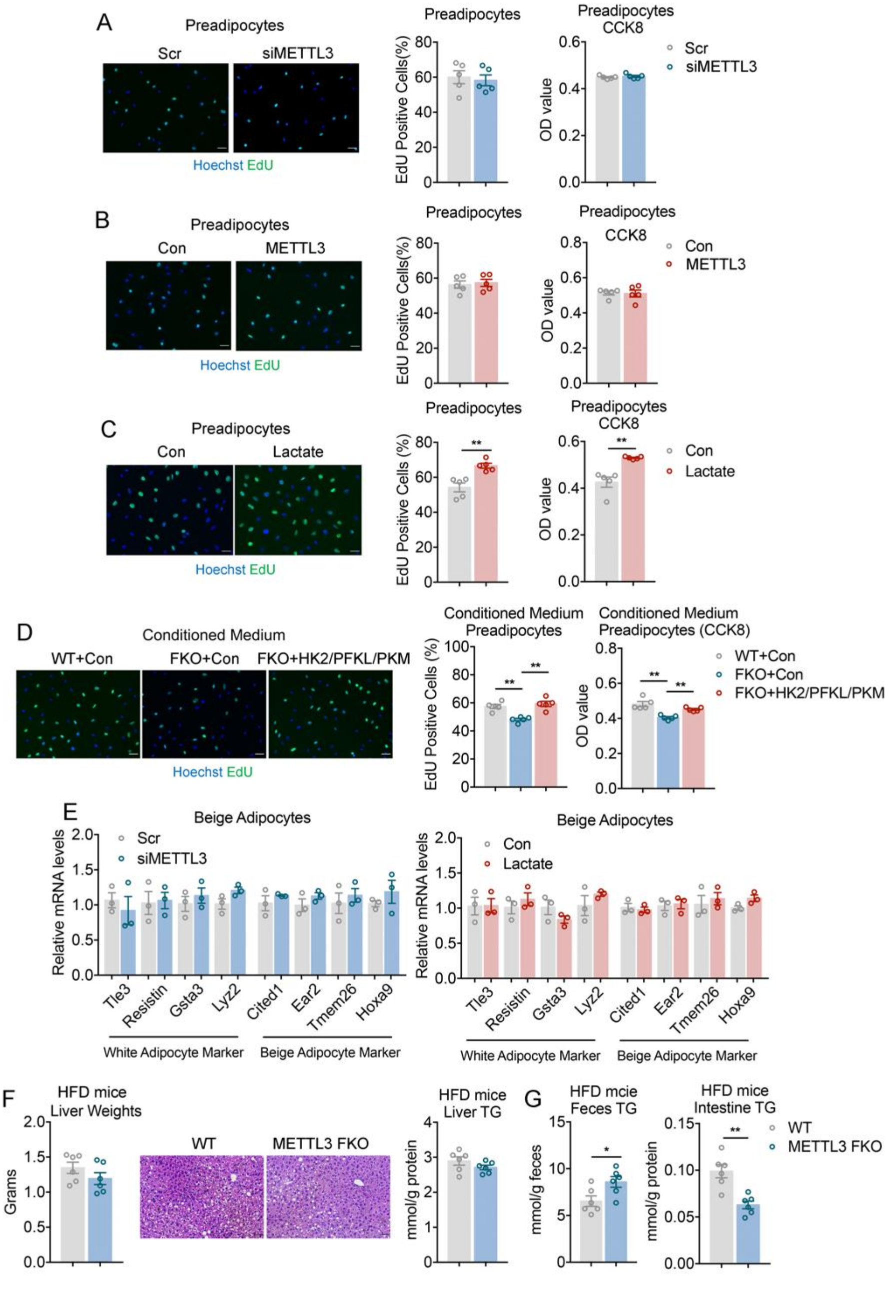


Figure S3

Supplementary Figure 3. Lactate promotes preadipocytes proliferation in vitro and liver metabolic characterizations of HFD-fed Mettl3 FKO mice.

A. EdU staining and CCK8 assay of preadipocytes treated with scramble (Scr) or Mettl3 knockdown (siMettl3) (n=5).

B. EdU staining and CCK8 assay of preadipocytes treated with Control (Con) or Mettl3 overexpression (Mettl3) (n=5).

C. EdU staining and CCK8 assay of preadipocytes treated with or without lactate (n=5) .

D. EdU staining and CCK8 assay of preadipocytes treated with conditioned medium from primary adipocytes of WT, Mettl3 FKO or Mettl3 FKO treated with lentivirus for Hk2/Pfkl/Pkm overexpression (n=5).

E. Relative mRNA levels of white adipocyte- and beige adipocyte-selective markers in beige adipocytes derived from Mettl3-knockdown preadipocytes or lactate-treated preadipocytes (n=3).

F. Liver weight, representative H&E staining of liver tissues and hepatic TG of WT and Mettl3 FKO mice on HFD (n=6).

G. Small intestinal absorption of lipids including intestinal TG levels and fecal TG levels in WT and Mettl3 FKO mice on HFD (n=6).

Data are shown as mean ± SEM. Statistical significance was analyzed by unpaired Student's t-test. *P < 0.05, **P < 0.01. Scale bars, 50μm.

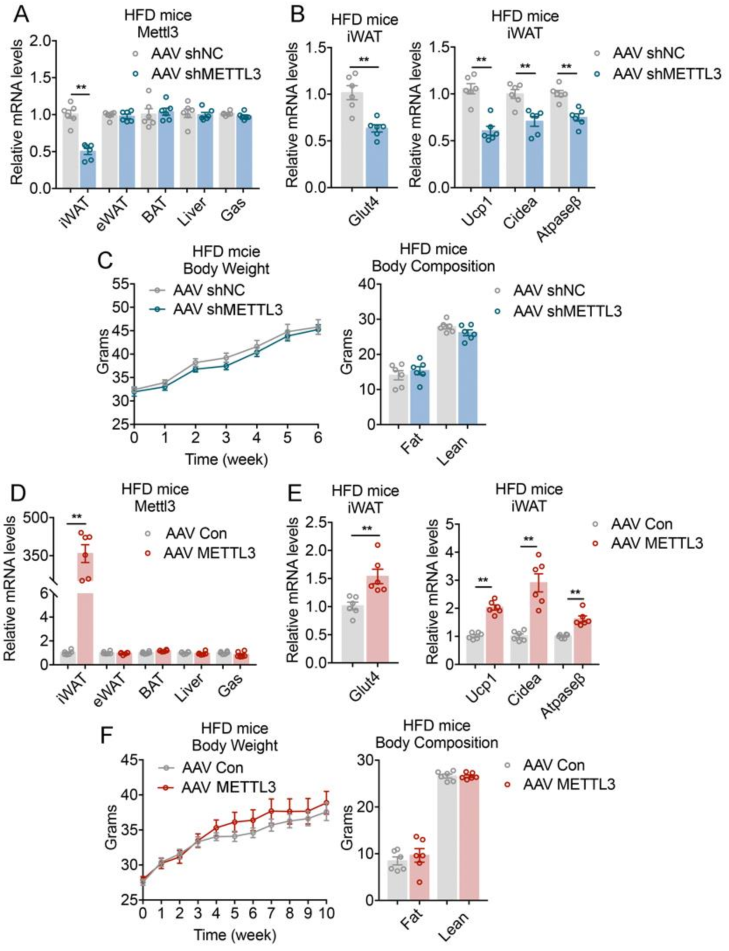


Figure S4

Supplementary Figure 4. Metabolic parameters of mice with Mettl3 deficiency or overexpression in iWAT.

A. Mettl3 expression in metabolic organs of AAV shNC and AAV shMettl3 mice on HFD for 6 weeks (n=6).

B. Relative Glut4 and thermogenesis mRNA levels in iWAT of AAV shNC and AAV shMettl3 mice on HFD (n=6).

C. Body weight (left) and body composition (right) of AAV shNC and

AAV shMettl3 mice on HFD for 6 weeks (n=6).

D. Mettl3 expression in metabolic organs of AAV Con and AAV Mettl3 mice on HFD for 10 weeks (n=6).

E. Relative Glut4 and thermogenesis mRNA levels in iWAT of AAV Con and AAV Mettl3 mice on HFD (n=6).

F. Body weight (left) and body composition (right) of AAV Con and AAV Mettl3 mice on HFD for 10 weeks (n=6).

Data are shown as mean ± SEM. Statistical significance was analyzed by unpaired Student's t-test. *$P < 0.05$, **$P < 0.01$.

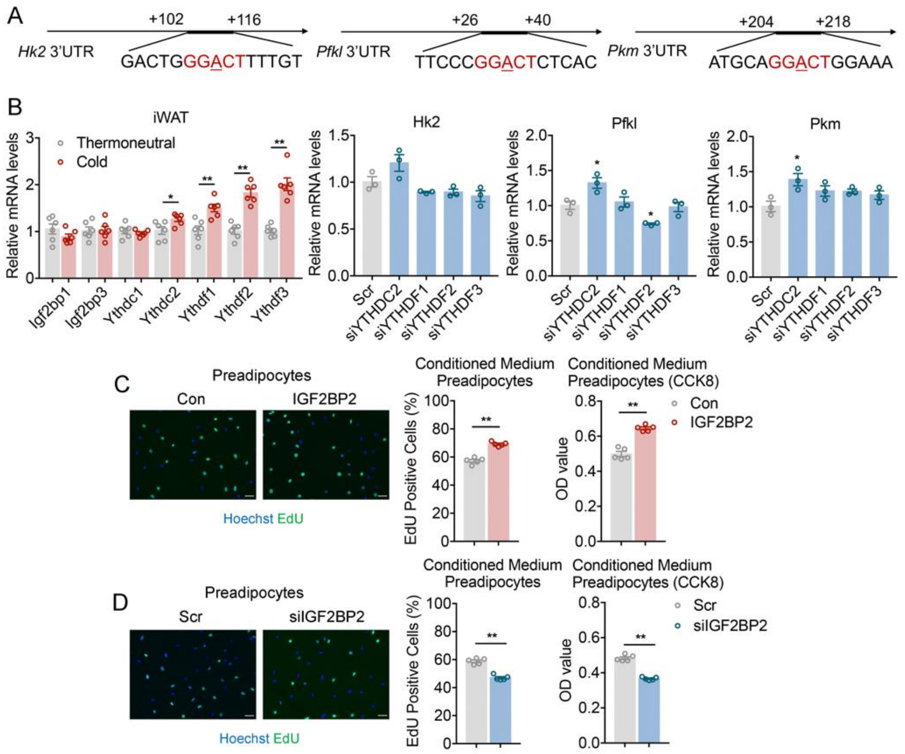


Figure S5

Supplementary Figure 5. Igf2bp2 induce preadipocyte proliferation in beige adipocytes.

A. The very high-confidence m6A site was identified in the 3'UTR region upon Hk2, Pfkl and Pkm mRNA based on the SRAMP software analysis.

B. Relative mRNA levels of various m6A reader including Igf2bp1/3, Ythdc1/2, Ythdf1/2/3 in iWAT from mice under chronic cold stimulation or thermoneutral condition (n=6); Relative mRNA levels of Hk2, Pfkl and Pkm in scramble (Scr) and Ythdc2, Ythdf1/2/3 knockdown (siYthdc2,

siYthdf11, siYthdf2 or siYthdf3) in immortalized beige adipocytes (n=3).

C. EdU staining and CCK8 assay of preadipocytes treated with conditioned medium from differentiated beige adipocytes with or without Igf2bp2 overexpression (n=5).

D. EdU staining and CCK8 assay of preadipocytes treated with conditioned medium from differentiated beige adipocytes with or without Igf2bp2 knockdown (n=5).

Data are shown as mean ± SEM. Statistical significance was analyzed by unpaired Student’s t-test. *P < 0.05, **P < 0.01.
